# Effect of information about the benefits and harms of mammography on women’s decision making: The InforMa randomised controlled trial

**DOI:** 10.1371/journal.pone.0214057

**Published:** 2019-03-26

**Authors:** María José Pérez-Lacasta, Montserrat Martínez-Alonso, Montse Garcia, Maria Sala, Lilisbeth Perestelo-Pérez, Carmen Vidal, Núria Codern-Bové, Maria Feijoo-Cid, Ana Toledo-Chávarri, Àngels Cardona, Anna Pons, Misericòrdia Carles-Lavila, Montserrat Rue, with the InforMa Group

**Affiliations:** 1 Department of Economics, University Rovira i Virgili, Reus, Spain; 2 Research Group on Statistics, Economic Evaluation and Health (GRAEES), Reus, Spain; 3 Research Centre on Industrial and Public Economics, (CREIP), Reus, Spain; 4 Department of Basic Medical Sciences, University of Lleida-IRBLLEIDA, Lleida, Spain; 5 Lleida Biomedical Research Institute (IRBLLEIDA), Lleida, Spain; 6 Cancer Prevention and Control Program, Catalan Institute of Oncology-IDIBELL, L’Hospitalet de Llobregat, Spain; 7 Department of Epidemiology and Evaluation, IMIM (Hospital del Mar Medical Research Institute), Barcelona, Spain; 8 Health Services Research on Chronic Patients Network (REDISSEC), Madrid, Spain; 9 Evaluation Unit of the Canary Islands Health Service (SESCS), Tenerife, Spain; 10 ÀreaQ, Evaluation and Qualitative Research, Barcelona, Spain; 11 Nursing and Occupational Therapy School (EUIT), Universitat Autònoma de Barcelona, Terrassa, Spain; 12 Department of Nursing, Faculty of Medicine, Universitat Autònoma de Barcelona, Bellaterra, Spain; 13 Grup de REcerca Multidisciplinar en SAlut i Societat (GREMSAS), Barcelona, Spain; 14 Canary Islands Foundation of Health Research (FUNCANIS), Tenerife, Spain; 15 Catalan Health Institut (ICS), Lleida, Spain; University of Texas MD Anderson Cancer Center, UNITED STATES

## Abstract

**Background:**

In Spain, women invited to breast screening are not usually informed about potential harms of screening. The objective of the InforMa study is to assess the effect of receiving information about the benefits and harms of breast screening on informed choice and other decision-making outcomes, in women approaching the age of invitation to mammography screening.

**Methods:**

Two-stage randomised controlled trial. In the first stage, 40 elementary territorial units of the public healthcare system were selected and randomised to intervention or control. In the second stage, women aged 49-50 years were randomly selected. The target sample size was 400 women. Women in the intervention arm received a decision aid (DA) with detailed information on the benefits and harms of screening. Women in the control arm received a standard leaflet that did not mention harms and recommended accepting the invitation to participate in the Breast Cancer Screening Program (BCSP). The primary outcome was informed choice, defined as adequate knowledge and intentions consistent with attitudes. Secondary outcomes included decisional conflict, worry about breast cancer, time perspective, opinions about the DA or the leaflet, and participation in the BCSP.

**Results:**

In the intervention group, 23.2% of 203 women made an informed choice compared to only 0.5% of 197 women in the control group (p < 0.001). Attitudes and intentions were similar in both study groups with a high frequency of women intending to be screened, 82.8% vs 82.2% (p = 0.893). Decisional conflict was significantly lower in the intervention group. No differences were observed in confidence in the decision, anxiety, and participation in BCSP.

**Conclusions:**

Women in Spain lack knowledge on the benefits and harms of breast screening. Providing quantitative information on benefits and harms has produced a considerable increase in knowledge and informed choice, with a high acceptance of the informative materials.

**Trial registration:**

Trial identifier NCT03046004 at ClinicalTrials.gov registry. Registered on February 4 2017. Trial name: InforMa study.

## Introduction

Breast cancer is the most frequent cancer among women in the world with nearly 1.7 million new cancer cases diagnosed in 2012 (25% of all cancers) [1]. It is the second leading cause of cancer death in developed regions (15.4%) after lung cancer. The aim of screening with mammography is to detect and treat breast cancer at its earliest stage. It is estimated that screening reduces breast cancer mortality by 20% and that one breast cancer death is prevented for every 235 women invited to screening for 20 years [2]. However, this benefit needs to be weighed against the harms of screening, in particular the risk of overdiagnosis [3]. Although there is uncertainty and high variability around this risk, it is estimated that 11% of breast cancer cases are overdiagnosed from a population perspective, and about 19% from the perspective of a woman invited to screening [2].

The concerns about overdiagnosis have emphasized the importance of providing information on the benefits and harms of screening with mammography, so that women can actively participate in decision-making and make an informed choice based on their values and preferences [4, 5]. This recommendation faces the barrier of generalised public enthusiasm towards screening, as a result of more than thirty years of promotional efforts to encourage participation in screening programs in order to avoid a late diagnosis of a life-threatening but potentially curable disease. In fact, participation rate has been considered an important component of the evaluation of a mammography screening program [6]. Furthermore, there is evidence that both the general population and health professionals tend to have biased expectations of the benefits and harms of health interventions. Thus, two systematic reviews by Hoffmann and Del Mar show that both women and health professionals overestimate the benefits and underestimate the harms of screening [7, 8]. In addition, women overestimate the risk of having breast cancer and most of them have not been informed of the screening harms. In the DECISIONS study [9], a representative sample of US adults who had faced screening decisions reported that healthcare providers often failed to provide balanced information, particularly about the cons of screening. Thus, paternalism is still considerable in decision-making about screening. This attitude may be due, in part, to social pressure to avoid medical error or a late diagnosis, but it is also due to the inertia of health professionals to adapt to the new evidence available.

Prior to undertaking this study, we performed a qualitative study with focus groups of women and health professionals aimed at testing and improving an informative leaflet to be used as a decision aid (DA) in the present study [10]. The leaflet follows the criteria of Stacey et al. to be considered a DA: providing information about options and associated benefits/harms, and helping clarify congruence between decisions and personal values [11].

Women positively valued receiving information regarding benefits and harms. Providing information on overdiagnosis generated confusion among women and controversy among professionals. Faced with the new information presented by the DA, the majority of women expressed the need for shared decision-making with their health providers. We also performed a systematic review on the impact of DAs, in women aged 50 and below facing the decision to be screened for breast cancer. The review showed that DAs increase adequate knowledge and informed decision, however, there was heterogeneity among the studies in confidence in the decision [12]. In the subgroup analysis of randomised controlled trials there was a significant decrease in confidence in the decision and in intention to be screened in the intervention group.

This study has largely been based on a study by Hersch et al., the first randomised controlled trial to evaluate the effects of a DA among women entering the age of screening [13]. They focused on assessing whether information on overdiagnosis improved the level of informed choice about screening in New South Wales, Australia, one of the pioneer countries in citizens’ participation in healthcare decisions. In their study, the standard information leaflet already described benefits and limitations without giving chances of outcomes nor mentioning overdiagnosis. In Spain, up to this point, women invited to breast screening are not usually informed about the potential harms of screening.

### Study objective

The objective of the InforMa study is to assess the effect of receiving detailed information about the benefits and harms of breast cancer screening on informed choice and other decision-making outcomes, in women approaching the age of invitation to mammography screening.

## Methods

### Study design and participants

The study was designed as a parallel two-stage randomised 1:1 controlled trial (RCT). In the first stage, elementary territorial units of the healthcare system named Basic Health Areas (BHAs) were stratified by socioeconomic level [14] and 40 of them were selected and randomised to intervention or control using computer-generated blocks of size two. In the second stage, random samples of 30 to 50 women within each BHA were obtained.

Four breast cancer screening programs (BCSPs) of the Spanish public health system participated in the study. The Spanish BCSPs follow the European Guidelines for Quality Assurance in Mammographic Screening and their indicators meet the required standards [15]. All women resident in Spain aged 50 to 69 years are invited to participate in the population-based screening program every 2 years. The participant BCSPs are managed by Hospital del Mar in Barcelona, the Cancer Prevention and Control Program of the Catalan Institute of Oncology, the Canary Islands Health Service, and the Lleida Health Region.

The target sample size was 10 women per BHA, a total of 400 women, 200 in the intervention and 200 in the control group. We assumed that 60% of women invited to participate would accept and 20% of the participants would be lost to follow-up. The random allocation sequence was generated by a statistician with no contact with the participants (MR). All selected women received a mailed invitation letter with a summary of the study objectives. In an interval of two weeks, trained interviewers, not aware of the women’s allocation, contacted them by phone, briefly described the study and determined eligibility. Interviewers invited the selected women using the phrase “a study about information on advantages and disadvantages of breast screening” without mentioning specific terms as false positive or overdiagnosis. The interviewers informed them that participation consisted of answering two questionnaires, either via web or by phone with the questionnaires being sent ahead of time by mail. Women who met the inclusion criteria and agreed to participate were asked for informed consent which was recorded orally. For women who chose answering by phone, the interviews followed a structured outline and were continuously monitored by the study team.

The inclusion criteria were: being a woman, aged 49-50 years, that in 2-4 months was going to be invited to participate in the screening program for the first time. Women were excluded if they had a personal history of breast cancer, difficulty speaking Spanish or Catalan, or cognitive impairment that prevented them from understanding or completing the materials based on the interviewer’s judgment during the first call or because a relative reported that the selected woman was not able to respond.

The study was approved by the Ethics Committees of the Arnau de Vilanova University Hospital in Lleida (approval number 19/2014), Parc de Salut Mar in Barcelona (2014/5998/I), Bellvitge University Hospital in Hospitalet (PR349/14), and by the Scientific and Ethics Committee of Nuestra Señora de la Candelaria University Hospital in Tenerife (Canary Islands, Spain). The published protocol describes with detail the study design and methods [14] (the trial study protocol approved by the Ethics Committee are included in S1 and S2 Files). The InforMa study was registered with the ClinicalTrials.gov registry, number NCT03046004. The methods and results are reported in accordance with the Consolidated Standards of Reporting Trials (CONSORT) Statement [16] (S3 File).

### Interventions

After acceptance, the pre-intervention questionnaire (Q1, S4 File) was sent to all study participants. Q1 included baseline demographics, previous screening experience, breast cancer risk factors, and general screening knowledge, attitudes, and intentions. After completion of Q1, women in the intervention arm received a DA that was a leaflet with detailed information on the benefits and harms of screening. The DA was developed and tested through the qualitative study above-mentioned. Details on the design of the DA can be found in Toledo-Chávarri et al. [10]. As usual in the Spanish BCSPs, women in the control arm received a standard leaflet that did not mention harms and recommended accepting the invitation to participate in the biennial exams of the BCSP. (The intervention DA and control leaflet are included in S5 and S6 Files, respectively).

The post-intervention questionnaire (Q2, S7 File) was planned to be completed at 2–4 weeks after the estimated leaflet delivery date (either intervention or control). Q2 included the questions needed to obtain the primary and secondary outcomes of the study. Participation in the screening program was assessed at 3 months after completing accrual.

Women that did not respond the questionnaires Q1 and Q2 in the planned period were re-called by phone or were sent weekly reminders via web up to a maximum of 5 times.

### Outcomes

With the aim of comparability, the outcome measures follow the Hersch et al. study protocol very closely [17]. Most of the outcome measures were obtained through validated scales that have shown suitability in previous studies. We translated them to Catalan and Spanish.

The primary outcome was informed choice about breast cancer screening, a dichotomous outcome defined as adequate knowledge and intentions consistent with attitudes (positive attitudes and intentions or negative attitudes and intentions) [13, 18–20]. Thus, informed choice combines three constructs, knowledge, attitudes and intentions that were obtained as explained below.

Conceptual and numerical knowledge was assessed following the Hersch et al. study [13] adapted to the mortality, incidence, and outcomes of screening data of our setting. A total of 22 marks could be obtained, 11 coming from ten questions on conceptual knowledge and 11 coming from four questions on numerical knowledge that measured absolute and relative values of the screening outcomes. In our study, however, we did not use open numerical questions (asking for absolute frequencies of the outcomes), but instead, we used multiple choice questions on frequency categories. We modified these questions based on the answers to the pilot study, where the frequencies of benefits were highly overestimated and the frequencies of harms highly underestimated. As in the Hersch et al. study, the threshold to define adequate knowledge for informed choice was to score at least 50% of the available marks, including at least one numerical mark, on all the three screening outcome subscales that refer to mortality reduction, overdiagnosis, and false positives.

Screening attitudes were measured using five items adapted from Dormandy et al. [21]. Total scores could range from 5 to 25. For informed choice, we set the threshold of a positive attitude at 20. Intention to participate in screening was measured with one question with five responses that, for informed choice, was dichotomized as categories 1–2 (responses definitely will and will) indicating ‘intending’ to screen and categories 3–5 (responses unsure, will not, and definitely will not), indicating ‘not intending’ to screen [22, 23].

Secondary outcomes (eg, decisional conflict, worry about breast cancer, time perspective, opinions about the DA, and participation in the BCSP) have been fully detailed in the protocol [14]. Decision conflict was assessed using the Decisional Conflict Scale (10-item low literacy version) by O’Connor [24, 25]. Anxiety about screening participation was measured with the six-item short form of the Spielberger State Trait Anxiety Inventory [26]. Time perspective was assessed using the short form of the Consideration of Future Consequences Scale [27]. The response categories and scores of the secondary outcomes are detailed in the Results section tables.

### Statistical analysis

To estimate the sample size we used the formula proposed by Donner and Klar for comparison of proportions in cluster randomization trials [28]. By consensus, we considered as clinically relevant an absolute difference of 20% in the primary outcome informed choice. Assuming that the proportion of one group was 50% (conservative scenario) and estimating an intraclass correlation coefficient equal to 0.1 (cluster sampling) and a maximum of 13 women per cluster, in order to achieve an 80% power to detect a group difference of 20%, with a two-sided significance level of 5%, a sample size of 200 women per group was required. The 400 women were distributed with 100 in each BCSP. This sample size was sufficient to detect a difference of 20% in the secondary outcome intention to participate and a mean difference of 0.35 standard deviations in the knowledge and attitudes scales. Assuming that 60% of women invited to participate would accept and 20% would be loss to follow-up, a minimum of 840 women, 210 per BCSP, were planned to be invited.

We performed the statistical analysis as planned in the protocol [14]. Primary and secondary outcomes in the two study groups were compared using the chi-square test for categorical variables and the Student’s t for quantitative variables. Both tests were adjusted by the clustering of responses within BHAs using the Rao-Scott correction [29]. Statistical significance and confidence intervals were obtained using the svytable and svyttest functions of the **survey** package [30], in the **R** language [31].

The proportion of women with an informed choice were compared between the two study groups. The three component variables of informed choice: knowledge, attitudes and intentions, were also analyzed and reported separately.

A mixed-effects model with an unstructured covariance matrix was used to assess the effect of the intervention on the primary outcome accounting for the women’s characteristics that showed imbalance at baseline.

The main analysis included all women that had completed the pre- and post-intervention questionnaires with no missing data. Missing values imputation was not done in women lost to follow-up or who had not completed the post-intervention questionnaire. A sensitivity analysis was performed including all women that had information on the main outcome, despite having missing data in the secondary outcomes.

All the data was entered and recorded with the open source LimeSurvey [32]. Range checks and error alerts were used to prevent invalid data. The **R** programming language (versions 3.4.2 and 3.4.3) [31] and the **RStudio** environment [33] were used for the data analysis.

## Results

**Fig 1:**
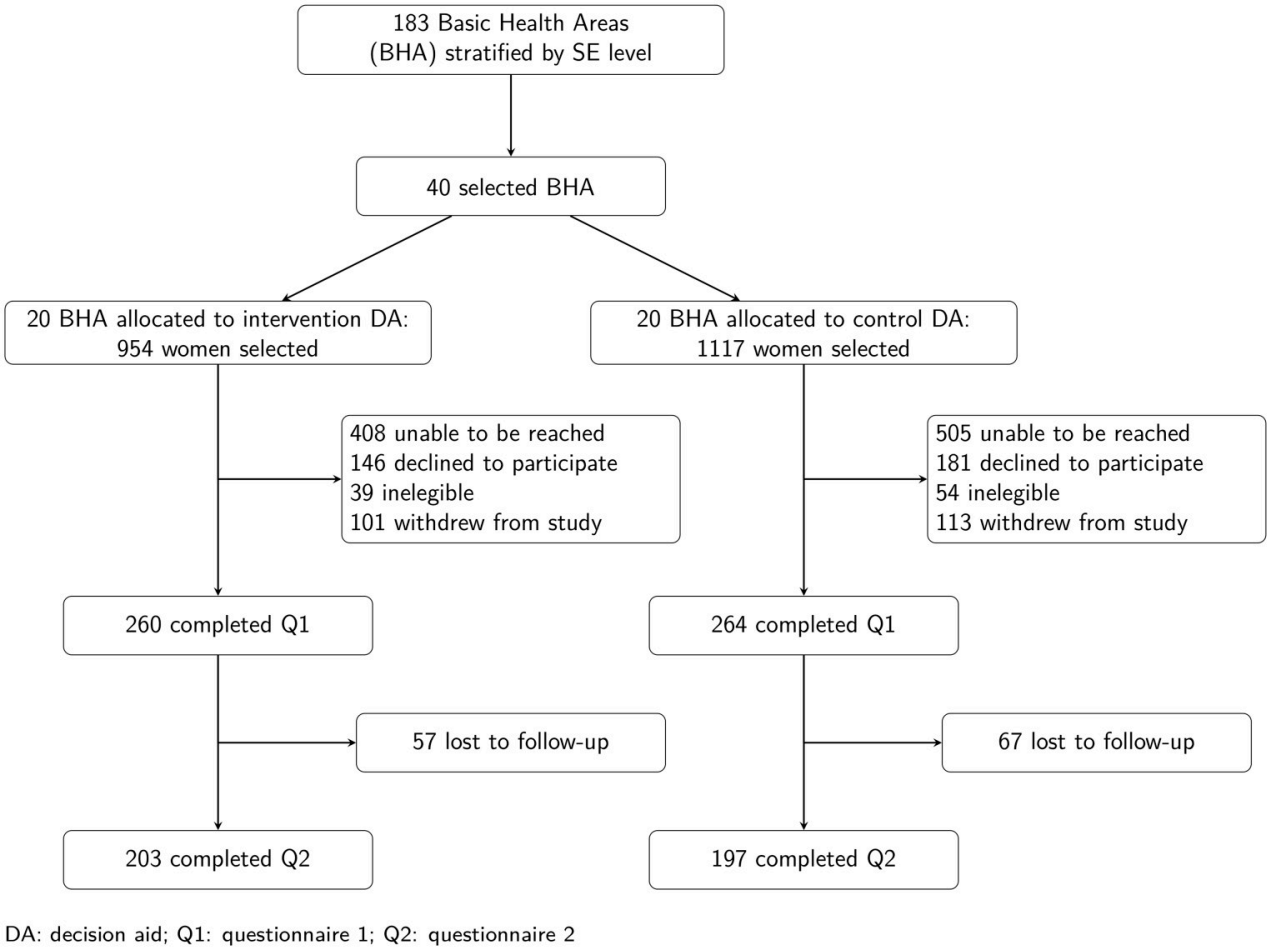
CONSORT flow diagram.

A total of 2071 women, 954 in the intervention group and 1117 in the control group were randomly selected within the 40 previously selected BHA. The CONSORT flow diagram of the progress through the phases of the study is depicted in Fig 1.

The fieldwork was conducted between July 1, 2016 and September 14, 2017. The trial ended when the sample size for the primary and secondary outcomes was achieved. Follow-up for assessment of participation in the BCSP was closed on June 20, 2018.

Trained interviewers were able to reach 1158 of the selected women, 546 and 612 in the intervention and control groups, respectively. The baseline questionnaire was completed by 524 women (49.2% of the invited and eligible), 260 and 264 women in the intervention and control groups, respectively. The post-intervention questionnaire was completed by 400 women, 203 in the intervention group and 197 in the control group, which represents a response rate of 37.6% among the invited and eligible women. Seven women with partial responses in the post-intervention questionnaire, 3 in the intervention group and 4 in the control group, had responded the primary outcome variables. The sensitivity analysis showed no differences with the complete case analysis in terms of baseline characteristics comparability or primary outcome. The results presented here refer to the 400 women that completed the study, 305 of which responded via web.

At baseline, women in both study groups were similar with respect to sociodemographic variables, family history of breast cancer, perceived knowledge on benefits and harms of breast cancer screening and opinions on breast screening participation (Table 1).

**Table 1:** Baseline characteristics of participants.

	Intervention n = 203	Control n = 197	Difference (95% CI)
**Demographics and health**			
Mean (SD) age (years)	50.14 (0.45)	50.19 (0.46)	-0.05 (-0.23, 0.13)
Education			
Less than secondary school graduation	36 (17.7%)	34 (17.3%)	
Secondary school diploma or equivalent	17 (8.4%)	24 (12.2%)	
Some postsecondary education	46 (22.7%)	55 (27.9%)	
Postsecondary certificate, diploma or degree	104 (51.2%)	84 (42.6%)	
Current employment			
No paid job	62 (30.5%)	45 (22.8%)	
Working	141 (69.5%)	152 (77.2%)	
Place of birth			
Catalonia	119 (58.6%)	115 (58.4%)	
Other places in Spain	61 (30%)	61 (31%)	
Other countries	23 (11.3%)	21 (10.7%)	
Number of children			
None	34 (16.7%)	37 (18.8%)	
One	50 (24.6%)	51 (25.9%)	
Two or more	119 (58.6%)	109 (55.3%)	
Family history of breast cancer	20 (9.9%)	17 (8.6%)	1.2 (-4.9, 7.4)
Previous use of mammograms	170 (83.7%)	149 (75.6%)	8.1 (-0.3, 16.5)
**General knowledge on breast screening**			
Means (SD) on perceived knowledge on benefits	3.91 (1.2)	3.81 (1.34)	0.1 (-0.13, 0.33)
Means (SD) on perceived knowledge on harms	3.18 (1.39)	3.14 (1.42)	0.04 (-0.22, 0.3)
Means (SD) on perceived overall knowledge	7.09 (2.29)	6.95 (2.52)	0.14 (-0.3, 0.58)
**Attitudes towards having breast screening** ^a^			
For you, knowing the benefits of breast screening is important	194 (95.6%)	180 (91.4%)	4.2 (0.5, 7.9)
For you, knowing the harms of breast screening is important	193 (95.1%)	176 (89.3%)	5.7 (1.7, 9.8)
Mean (SD) attitude on benefits	4.79 (0.53)	4.59 (0.75)	0.19 (0.07, 0.32)
Mean (SD) attitude on harms	4.74 (0.62)	4.54 (0.82)	0.2 (0.06, 0.34)
Mean (SD) overall attitude score	9.53 (1.04)	9.14 (1.49)	0.39 (0.15, 0.64)
**On breast screening participation** ^b^			
For you, screening participation is right or very right	167 (82.3%)	166 (84.3%)	-2 (-8.9, 4.9)
For you, screening participation is important or very important	174 (85.7%)	170 (86.3%)	-0.6 (-6.5, 5.4)
For you, screening participation is unpleasant or very unpleasant	32 (15.8%)	29 (14.7%)	1 (-6.3, 8.4)
Mean (SD) on screening is right	4.33 (0.92)	4.39 (0.88)	-0.05 (-0.22, 0.12)
Mean (SD) on screening is important	4.44 (0.79)	4.42 (0.87)	0.02 (-0.11, 0.15)
Mean (SD) on screening is unpleasant (reverse)	3.97 (1.32)	4.04 (1.22)	-0.07 (-0.34, 0.21)
Mean (SD) overall	12.75 (2.21)	12.84 (2.3)	-0.09 (-0.55, 0.36)

^a^ Attitude items were rated on a scale from not at all important (1) to very important (5). Overall scores could range from 2 to 10: higher scores indicate more positive attitudes.

^b^ Participation items were rated on a scale from not at all (1) to very much (5). Overall scores could range from 3 to 15: higher scores indicate more positive attitude towards participation.

Previous use of mammograms was higher in the intervention group (83.7% versus 75.6%), with a nearly significant p-value = 0.052. More than 85% of the women in both groups considered that screening participation is important or very important. Statistically significant differences were observed in attitudes towards importance of knowing the benefits and harms of breast screening (p = 0.029 and 0.011, respectively), both slightly higher in the intervention group. Mean attitude scores on benefits, harms and overall were also significantly higher in the intervention group than in the control group, at baseline.

Table 2 presents the results for the main outcome (informed choice), and its components (knowledge, attitudes and intentions).

**Table 2:** Analysis of primary outcome.

	Intervention n = 203	Control n = 197	Difference (95% CI)	p-value ^a^
**Informed choice** ^b^				
Made an informed choice	47 (23.2%)	1 (0.5%)	22.6 (17.1, 28.2)	< 0.001
**Adequate knowledge (conceptual and numerical items combined)**				
Breast cancer mortality benefit	111 (54.7%)	41 (20.8%)	33.9 (24.6, 43.2)	< 0.001
False positives	86 (42.4%)	12 (6.1%)	36.3 (28.3, 44.2)	< 0.001
Overdiagnosis	110 (54.2%)	16 (8.1%)	46.1 (38.2, 53.9)	< 0.001
Adequate knowledge across all three subscales	68 (33.5%)	2 (1%)	32.5 (25.5, 39.4)	< 0.001
**Knowledge (conceptual items individually)** ^c^				
Screening is for women without symptoms	182 (89.7%)	167 (84.8%)	4.9 (-1.7, 11.4)	0.143
Screening reduces breast cancer deaths (benefit)	190 (93.6%)	189 (95.9%)	-4.7 (-12.8, 3.4)	0.25
Screening will not find every breast cancer (benefit)	141 (69.5%)	76 (38.6%)	30.9 (20.7, 41.1)	< 0.001
Screening may lead to false positive results (false positives)	202 (99.5%)	188 (95.4%)	4.1 (0.8, 7.3)	0.006
Screening increases breast cancer diagnoses (overdiagnosis)	171 (84.2%)	146 (74.1%)	10.1 (2, 18.3)	0.016
Overdiagnosis vs false positives distinction (overdiagnosis)	78 (38.4%)	27 (13.7%)	24.7 (17.1, 32.3)	< 0.001
Not all breast cancers cause illness and death (overdiagnosis)	119 (58.6%)	49 (24.9%)	33.7 (24.5, 42.9)	< 0.001
Cannot predict if a cancer will cause harm (overdiagnosis)	132 (65%)	70 (35.5%)	29.5 (17.9, 41.1)	< 0.001
Cancer that might not cause problem is treated (overdiagnosis)	176 (86.7%)	146 (74.1%)	12.6 (3.1, 22.1)	0.005
Some women get treatment they do not need (overdiagnosis)	118 (58.1%)	39 (19.8%)	38.3 (29, 47.7)	< 0.001
Overdiagnose more often than prevent death (overdiagnosis)	123 (60.6%)	90 (45.7%)	14.9 (4.4, 25.4)	0.006
**Attitudes towards having breast screening** ^d^				
For you, having breast screening is…				
Beneficial	4.47	4.63	-0.16 (-0.29, -0.02)	0.032
Harmful (reverse scored)	3.51	3.42	0.1 (-0.15, 0.34)	0.453
A good thing	4.43	4.47	-0.03 (-0.19, 0.12)	0.669
Important	4.54	4.62	-0.08 (-0.22, 0.07)	0.296
Worthwhile	4.53	4.63	-0.1 (-0.23, 0.02)	0.115
Mean (SD) overall attitudes score	21.49 (3.63)	21.77 (3.33)	-0.27 (-0.85, 0.3)	0.357
Positive attitudes to screening (scores >= 20)	154 (75.9%)	155 (78.7%)	-2.8 (-11.8, 6.2)	0.544
Most positive (scores 24-25)	75 (36.9%)	77 (39.1%)		0.279
Scores 19-23	89 (43.8%)	93 (47.2%)		
Scores 14-18	33 (16.3%)	20 (10.2%)		
Scores 5-13	6 (3%)	7 (3.6%)		
**Intentions about having breast screening**				
Intending to be screened (definitely or likely)	168 (82.8%)	162 (82.2%)	0.5 (-7.1, 8.2)	0.893
Definitely will	114 (56.2%)	115 (58.4%)		0.928
Likely to	54 (26.6%)	47 (23.9%)		
Unsure	25 (12.3%)	24 (12.2%)		
Not likely to, or definitely will not	10 (4.9%)	11 (5.6%)		

^a^ The Chi-square tests for categorical variables and the Student’s t tests for quantitative variables were adjusted for clustering using the Rao-Scott correction.

^b^ Informed choice was defined as adequate knowledge and intentions consistent with attitudes (positive or negative).

^c^ Conceptual knowledge subscales were for benefit, false positives, and overdiagnosis.

^d^ Attitude items were rated from strongly disagree (1) to strongly agree (5). Overall scores could range from 5 to 25: higher scores indicate more positive attitudes.

In the intervention group, 47 (23.2%) of 203 women were judged to have made an informed choice compared to only 1 (0.5%) of 197 women in the control group (difference 22.6 (17.1, 28.2), p < 0.001). When conceptual and numerical knowledge items were combined, 33.5% of women in the intervention group had adequate knowledge across the three subscales (benefit, false positives and overdiagnosis) compared to 1% in the control group (p<0.001). The highest difference was observed in overdiagnosis, 46.1% (38.2, 53.9) p<0.001, followed by false positives and mortality reduction. Knowledge on conceptual items showed statistically significant differences among both study groups except on the purpose of screening (mortality reduction) and that screening is for women without symptoms. The highest difference among groups was on the overdiagnosis conceptual items, with 38.3% (29.0, 47.7) for the *Some women get treatment they do not need* statement.

When mixed effects models for the primary outcome were fitted to adjust for the baseline differences observed in the attitudes towards screening, the effect estimates did not change. The models’ results are not presented here.

Table 3 shows the mean scores and their differences in all the conceptual and numerical knowledge subscales by study group. The highest differences were observed in the numerical subscales with a better understanding of the benefits and harms of screening in the intervention group.

**Table 3:** Mean scores on knowledge subscales.

	Marks available	Intervention n = 203	Control n = 197	Difference (95% CI)	p-value ^a^
**Knowledge subscale**					
Breast cancer mortality benefit					
Conceptual	3	2.57	2.3	0.26 (0.13, 0.4)	< 0.001
Numerical	5	2.38	0.85	1.53 (1.16, 1.9)	< 0.001
Total	8	4.95	3.15	1.79 (1.38, 2.2)	< 0.001
False-positive screening results					
Conceptual	1	1	0.95	0.04 (0.01, 0.07)	0.019
Numerical	3	1.34	0.35	0.99 (0.75, 1.24)	< 0.001
Total	4	2.34	1.3	1.04 (0.78, 1.29)	< 0.001
Overdiagnosis					
Conceptual	7	4.52	2.88	1.64 (1.35, 1.93)	< 0.001
Numerical	3	1.49	0.49	1 (0.78, 1.22)	< 0.001
Total	10	6.01	3.37	2.64 (2.24, 3.04)	< 0.001
**All subscales**					
Conceptual	11	8.08	6.14	1.94 (1.56, 2.32)	< 0.001
Numerical	11	5.22	1.69	3.53 (2.78, 4.27)	< 0.001
Total	22	13.3	7.83	5.47 (4.5, 6.44)	< 0.001

^a^ The Student’s t tests for quantitative variables were adjusted for clustering using the Rao-Scott correction.

**Fig 2:**
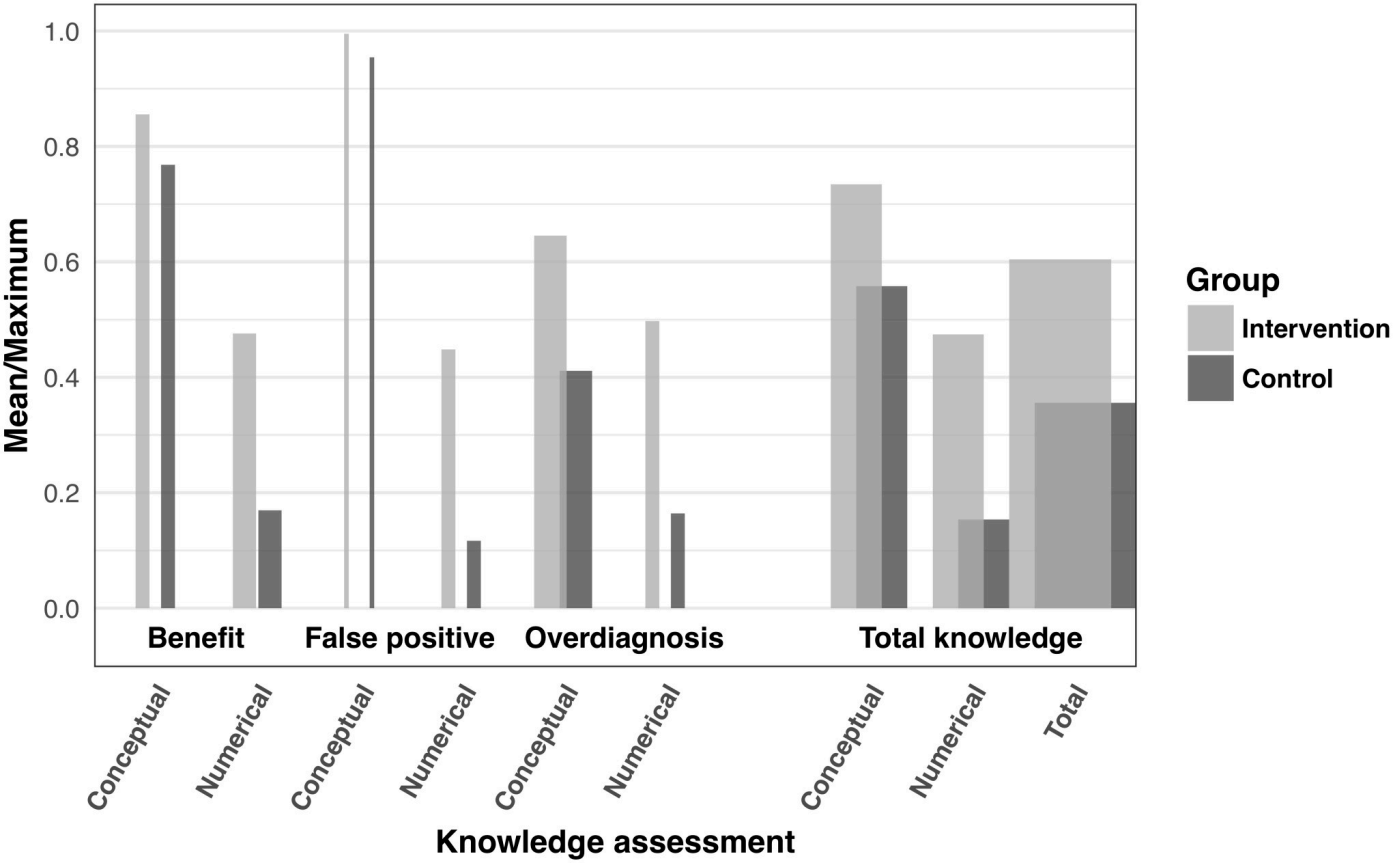
Relative mean scores on knowledge subscales, with respect to the maximum available score. Bars width indicate the contribution of the available marks for each subscale to the total available marks.

Fig 2 expresses knowledge in relative terms and displays observed mean scores divided by their corresponding maximum available marks, specified in Table 3.

The highest and lowest relative scores, in both study groups, correspond to the conceptual and numerical knowledge of a false positive result, respectively. When considering all subscales, conceptual knowledge achieves higher relative values than numerical knowledge, which is around 50% in the intervention group and 15% in the control group.

After the intervention, attitudes towards having breast screening were similar in both study groups (Table 2). Women in the control group had a slightly higher score in *having breast screening is beneficial* (p = 0.032). A positive attitude (score >= 20) was expressed by 75.9% of women in the intervention group vs 78.7% in the control group (p = 0.544). Intentions about having breast screening were very similar in both study groups, with a high frequency of women intending to be screened *definitely or likely*, 82.8% vs 82.2% (p = 0.893).

Table 4 presents the secondary outcomes.

**Table 4:** Analysis of secondary outcomes.

	Intervention n = 203	Control n = 197	Difference (95% CI)	p-value ^a^
**Decisional confict** ^b^				
Mean score	13.77 (18.55)	18.53 (20.25)	-4.76 (-8.52, -1)	0.018
0	88 (43.3%)	72 (36.5%)		0.006
1-24	66 (32.5%)	48 (24.4%)		
>= 25	49 (24.1%)	77 (39.1%)		
Mean uncertainty subscore	11.33	12.06	-0.73 (-4.77, 3.32)	0.727
Mean informed subscore	18.56	28.26	-9.7 (-15.5, -3.9)	0.002
Mean values clarity subscore	14.16	18.02	-3.86 (-9.1, 1.38)	0.157
Mean support subscore	10.34	13.45	-3.11 (-6.75, 0.54)	0.103
**Confidence in decision-making** ^c^				
Mean score	4.23 (0.83)	4.2 (0.86)	0.02 (-0.12, 0.16)	0.761
**Anxiety** ^d^				
Mean score	34.94 (12.75)	34.13 (14.54)	0.81 (-2.26, 3.89)	0.607
Worry about breast cancer				
Not worried at all	66 (32.5%)	63 (32%)		0.879
A bit worried	93 (45.8%)	95 (48.2%)		
Quite worried or very worried	44 (21.7%)	39 (19.8%)		
**Anticipated regret**				
Might later regret if do not screen				
Strongly agree	85 (41.9%)	90 (45.7%)		0.733
Agree	68 (33.5%)	65 (33%)		
Neither agree nor disagree	46 (22.7%)	37 (18.8%)		
Disagree or strongly disagree	4 (2%)	5 (2.5%)		
Might later regret if do screen				
Strongly agree or agree	14 (6.9%)	21 (10.7%)		0.246
Neither agree nor disagree	49 (24.1%)	40 (20.3%)		
Disagree	77 (37.9%)	65 (33%)		
Strongly disagree	63 (31%)	71 (36%)		
**Temporal orientation** ^e^				
Mean score	14.18 (3.07)	13.85 (3.04)	0.33 (-0.3, 0.96)	0.31
In deciding whether to have screening, how important is it for you to consider the chance of…				
Avoiding death from breast cancer				
Very important	169 (83.3%)	161 (81.7%)		0.232
Quite important	32 (15.8%)	31 (15.7%)		
A bit important	0 (0%)	4 (2%)		
Not at all important	2 (1%)	1 (0.5%)		
Overdiagnosis				
Very important	95 (46.8%)	98 (49.7%)		0.74
Quite important	80 (39.4%)	79 (40.1%)		
A bit important	23 (11.3%)	17 (8.6%)		
Not at all important	5 (2.5%)	3 (1.5%)		
False positives				
Very important	114 (56.2%)	106 (53.8%)		0.142
Quite important	58 (28.6%)	70 (35.5%)		
A bit important	20 (9.9%)	18 (9.1%)		
Not at all important	11 (5.4%)	3 (1.5%)		
**Perceived risk**				
Perceived risk of breast cancer				
No chance	18 (8.9%)	17 (8.6%)		0.937
Low chance	71 (35%)	66 (33.5%)		
Medium or high chance	114 (56.2%)	114 (57.9%)		
Perceived risk of breast cancer relative to the average woman				
Much lower	8 (3.9%)	11 (5.6%)		0.268
A bit lower	26 (12.8%)	15 (7.6%)		
About the same	140 (69%)	145 (73.6%)		
A bit higher or much higher	29 (14.3%)	26 (13.2%)		
Compared with the average screened woman, if you are screened, how likely is it that you would…				
Avoid dying from breast cancer				
Much less likely	28 (13.8%)	34 (17.3%)		0.189
A bit less likely	49 (24.1%)	57 (28.9%)		
About the same	32 (15.8%)	34 (17.3%)		
A bit more likely	61 (30%)	38 (19.3%)		
Much more likely	33 (16.3%)	34 (17.3%)		
Experience overdiagnosis				
Much less likely	16 (7.9%)	21 (10.7%)		0.019
A bit less likely	17 (8.4%)	38 (19.3%)		
About the same	64 (31.5%)	61 (31%)		
A bit more likely	85 (41.9%)	61 (31%)		
Much more likely	21 (10.3%)	16 (8.1%)		
Have a false positive				
Much less likely	13 (6.4%)	15 (7.6%)		0.035
A bit less likely	22 (10.8%)	35 (17.8%)		
About the same	67 (33%)	78 (39.6%)		
A bit more likely	76 (37.4%)	56 (28.4%)		
Much more likely	25 (12.3%)	13 (6.6%)		
**Participated in the screening program**	128 (63.1%)	129 (65.5%)	-2.4 (-17.1, 12.2)	0.746

^a^ The Chi-square tests for categorical variables and the Student’s t tests for quantitative variables were adjusted for clustering using the Rao-Scott correction.

^b^ Decisional conflict was assessed using the Decisional Conflict Scale (10-item low literacy version) on a scale from 0 (no decisional conflict) to 100 (extreme decisional conflict). Scores less than 25 are associated with implementing decisions; score exceeding 37.5 are associated with decision delay or feeling unsure about implementation.

^c^ Confidence in decision-making, three items rated from 1 (not at all confident) to 5 (very confident).

^d^ State trait anxiety inventory (short form), on a scale from 20 to 80, with higher scores indicating greater levels of anxiety.

^e^ Consideration of future consequences scale (short form), on a scale from 4 to 20, with higher scores indicating a long-term time perspective (ie, greater orientation towards the future).

Decisional conflict was significantly lower in the intervention group, with an almost five point difference with respect to the control group in a 0-100 scale (p = 0.018), and 24.1% vs 39.1% of women with high decisional conflict (score >= 25). The highest difference was observed in the informed subscore, -9.7 points (-15.5, -3.9), p = 0.002, which was lower in the intervention group. No differences were observed in confidence in decision-making with high scores in both study groups, with means around 4.2 points for a maximum of 5 points (p = 0.761). There was no difference either among the study groups in the STAI anxiety score, with levels 35 and 34 (p = 0.607), for a score range 20-80. No differences were observed among the study groups in anticipated regret, with three out of four women considering that might later regret if do not screen. In temporal orientation, no differences among groups were observed either, with almost all women stating that avoiding death from breast cancer was very or quite important when deciding whether to have screening. Perceived risk of breast cancer was also similar in both study groups, with the majority of women having a medium or high perceived risk of breast cancer (56.2% vs 57.9%). When asked about the likelihood of experiencing benefits or harms, in relation to the average screened women, women in the intervention group thought that their chances of experiencing overdiagnosis or false positive results were higher than women in the control group. The intervention did not affect participation in the screening exam offered by the BCSP, 63.1% in the intervention group vs 65.5% in the control group, p = 0.746. As a exploratory analysis we assessed participation according to having made or not an informed choice. In the intervention group, women that made an informed choice, had lower participation in the BCSP than women without informed choice, 53.2% vs 66.0%. And, interestingly, women in the control group or in the intervention group without informed choice had similar BCSP participation rates.

Table 5 displays the assessment and acceptability of the leaflets by study group.

**Table 5:** Use and acceptability of the intervention decision aid and control leaflet.

	Intervention n = 203	Control n = 197	p-value ^a^
**Read leaflet**			
Read leaflet all the way through			
Yes	197 (97%)	194 (98.5%)	0.317
No	6 (3%)	3 (1.5%)	
**Length of decision aid**			
A little too long or much too long	25 (12.3%)	12 (6.1%)	0.008
Just about right	168 (82.8%)	164 (83.2%)	
Much too short or a little too short	10 (4.9%)	21 (10.7%)	
**Balance of leaflet**			
Clearly slanted towards screening	54 (26.6%)	84 (42.6%)	< 0.001
A little slanted towards screening	34 (16.7%)	28 (14.2%)	
Completely balanced	96 (47.3%)	84 (42.6%)	
A little slanted away from screening	17 (8.4%)	0 (0%)	
Clearly slanted away from screening	2 (1%)	1 (0.5%)	
**Leaflet was clear and easy to understand**			
Strongly agree	77 (37.9%)	108 (54.8%)	0.002
Agree	107 (52.7%)	78 (39.6%)	
Neither agree nor disagree	14 (6.9%)	10 (5.1%)	
Disagree or strongly disagree	5 (2.5%)	1 (0.5%)	
**Found leaflet helpful in making decision**			
Strongly agree	54 (26.6%)	63 (32%)	0.076
Agree	101 (49.8%)	107 (54.3%)	
Neither agree nor disagree	39 (19.2%)	22 (11.2%)	
Disagree or strongly disagree	9 (4.4%)	5 (2.5%)	

^a^ The Chi-square tests for categorical variables were adjusted by the clustering using the Rao-Scott correction.

Four out of five women in each group judged the leaflets length to be right. There were more women in the intervention group that found the leaflet a little or too long (12.3% vs 6.1%). There were statistically significant differences in the assessment of balance in the DA. Both study groups had a similar proportion of women that found the leaflets completely balanced (47.3% vs 42.6%). Nevertheless, more women in the control group judged their leaflet clearly slanted towards screening, 42.6% vs 26.6%, and reciprocally, more women in the intervention group judged their leaflet a little or clearly slanted away from screening (9.4% vs 0.5%). Both leaflets were judged clear and easy to understand with a higher proportion of women that strongly agreed on this point in the control group. Finally, most women in both groups found the leaflets helpful in making a decision (p = 0.076), with a higher proportion of women that agreed or strongly agreed in the control group. A low proportion of women, 4.4% in the intervention group and 2.5% in the control group, disagreed or strongly disagreed with the usefulness of the leaflet for making a decision on screening.

## Discussion

This study shows that knowledge and informed choice increased markedly when women received a DA containing explanatory and quantitative information on benefits and harms of breast cancer screening, compared to a leaflet that recommended screening participation, did not contain quantitative information on screening benefits and did not mention potential screening harms (overdiagnosis and false positives). Only one woman in 197 (0.5%) made an informed choice in the control group compared to one in four in the intervention group. Our study also shows that women in our study population are not aware of screening harms, specially overdiagnosis, which prevents them from making an informed choice. The highest difference among the study groups was found in knowledge about overdiagnosis, with 46 percentual points of difference between the intervention and control groups. Attitudes towards and intentions about having breast screening were similar in both study groups. Three out of four women had a positive attitude towards screening and four out of five expressed their intention about having breast screening. Thus, the information about adverse effects did not seem to affect the intention to participate in screening, as shown in previous research [34–36]. In our study, decisional conflict was significantly lower in the intervention group and no differences were observed in confidence in decision-making, anxiety, anticipated regret, temporal orientation, and perceived risk of breast cancer. However, women in the intervention group were more pessimistic about their chances of experiencing overdiagnosis or false positive results than women in the control group.

As Hersch et al. point out, the primary outcome reflects international commitments to informed choice as a key quality indicator [13]. We also valued their selection of validated instruments for primary and secondary outcomes and the opportunity of obtaining comparable results and facilitating future systematic reviews. With respect to the primary outcome, our results on informed choice were similar to their results for the intervention group, 23% versus 24% of women, but differed for the control group, 0.5% vs 15%. This difference can be due, in part, to differences in the informative leaflets provided to the control group. In the Hersch et al. study, the control leaflet was more balanced and informative than most screening program leaflets. Instead, our control leaflet reflects the current practice in our country, which consists of recommending screening for its benefits with no mention of the potential harms. Not only differences in the informative leaflets, but also cultural differences among study populations may explain the observed differences. Australia is one of the countries where citizens’ participation prevails, either in decisions that affect their health or their communities. An example of this popular involvement are citizens juries in health policy decisions [37]. A recently published study, conducted in Germany by Reder et al., with informed choice as the main outcome, showed that a DA resulted in a greater proportion of informed choices, a higher knowledge level, and less decisional conflict [38]. This study compared an information brochure with quantitative information on positive and negative screening results, including overdiagnosis, presented as absolute numbers in text (control), with a DA that contained absolute numbers supported by pictograms and a values clarification exercise (intervention). Informed choice for the control group, around 30% in the German study, was higher than informed choice for the intervention groups in the Hersch et al. [13] and our studies. And, informed choice for the intervention group increased to 62% and 40% at the post-intervention and follow-up (three months) time points.

In agreement with Hersch et al., conceptual knowledge was markedly higher than numerical knowledge in both study groups. If only conceptual items had been used, the proportions of women with adequate knowledge and therefore with informed choice would have been much higher. The low scores in numerical knowledge, that contributed to the low percentages of informed choice, can be a consequence of the overestimation of benefits and underestimation of harms that Hoffmann and Del Mar and others have reported [7, 39]. As mentioned in the Methods section, based on the pilot study, we decided to convert the open numerical questions to multiple choice questions. This modification oriented the response to the numerical evaluation part of the questionnaire, and consequently the proportion of women with adequate knowledge increased. Moreover, as Hersch et al. point out, the fact that currently no consensus exists on what constitutes knowledge suggests an important topic for future research [13].

With respect to secondary outcomes, providing information on the outcomes of screening may cause a certain level of decisional conflict, anxiety or reluctance to accept information on harms such as overdiagnosis, especially in women who are informed for the first time and have received messages about the importance of cancer screening over the years [35]. The fact that women in our study seem to find the control leaflet more helpful can be explained by doubts or concerns that the DA could have caused in women of the intervention group. Our systematic review on the effects of DAs did not find significant effects of DAs for decisional conflict, decision confidence and positive attitudes towards screening [12]. Nevertheless, in the subgroup of RCTs, there was a significant decrease in confidence in the decision and in intention to be screened. In our study, the intervention did not affect the attitudes towards screening, the intention to be screened, or the confidence in the decision, but in contrast to the Hersch et al. study, women that received the intervention DA expressed lower levels of decisional conflict than women in the control group, due to knowing the options and the benefits and side effects of each option. This result is consistent with the Cochrane review on DAs [11] which concludes that compared to usual care across a wide variety of decision contexts, people exposed to decision aids feel better informed and clearer about their values, experience lower decisional conflict, and probably have a more active role in decision-making. Our findings on decisional conflict are also consistent with those of Reder et al. [38], which differ from ours with respect to attitudes and intentions. Whereas, overall, we did not see any effect of the DA on attitudes and intentions, both Hersch et al. [13] and Reder et al. [38] found that fewer women in the intervention group expressed positive attitudes towards screening and fewer women intended to be screened. Our finding that women who made an informed choice had a lower participation in breast screening would be in line with the studies mentioned.

Among the strengths of our study, this is the first RCT in Spain evaluating the effects of providing information on the benefits and harms of screening with mammography prior to the first invitation. We also consider it a strength to have provided evidence on the low level of knowledge that women in our country have on the outcomes of screening. This is an unexpected result considering that about 80% of the study participants reported previous use of opportunistic mammograms, which are the mammograms offered by a doctor or health professional outside of an organised screening program. Our results on the acceptance of the DA, on the intention to participate in breast screening, and on the lower level of decisional conflict for women in the intervention group indicate that providing information on the screening harms is justified and should not scare health professionals. We expect that providing information on the existence and magnitude of benefits and adverse effects of screening will increase informed choice and empowerment for women in our country. Nevertheless, an open debate still exists about the best way to communicate the concept of overdiagnosis to a non-specialised public because the concept itself is difficult to understand [13, 36].

Our study also has some limitations. First, only 56% of women in the initially selected sample could be reached and only around 38% of those invited completed the study. Thus, recruitment or dropout biases may limit, to some extent, the generalisation of our results to the target screening population. Second, we have assessed the effect of informing women by sending them a DA before the invitation to be screened. Nevertheless, for many women, decisions on screening would benefit from a shared decision-making process, where they could ask and interact with their healthcare providers. Currently, we are carrying out a feasibility study on shared decision-making in personalised risk-based screening. Third, our study design assessed the short-term impact of the intervention. A longer follow-up and longitudinal outcomes would have made it possible to assess knowledge decay or retention, and long-term impact on screening participation. Four, we have not evaluated whether women’s characteristics, such as educational level or employment status, modify the effect of receiving information on informed choice or on participation in the screening program. These analyses or other more complex studies, such as the recent work of Hersch et al. [40] that explores the psychological pathways involved in how information is processed and how this influences decisions, may help to develop more effective communication tools and decision support resources. Finally, except for some instruments that had a Spanish version, like the O’Connor scale, the majority of items in our questionnaires were translated from the Hersch et al. study to Spanish and Catalan by members of the research team with a deep knowledge of the research area and piloted in non-participant selected women. Although we did not perform a cross-cultural adaptation/validation, given that most of the original questions were short, precise and formulated in simple language, we think that the results would have not changed much if a panel of experts had performed this task.

## Conclusion

In conclusion, our study indicates that women in Spain lack knowledge on the benefits and harms of breast cancer screening. Providing quantitative information on benefits and harms has produced a considerable increase in knowledge and informed choice, together with a high acceptance of the informational materials and lower decisional conflict than women in the control group. Therefore, our results highlight the importance of leaving behind paternalistic attitudes and assuming the ethical responsibility to inform women appropriately and help them to decide according to their values and preferences.

## Supporting information

S1 File Trial study protocol approved by the Ethics Committee. (PDF)

S2 File Translated study protocol. (PDF)

S3 File CONSORT checklist. (DOC)

S4 File Pre-intervention questionnaire, Q1. (PDF)

S5 File Decision aid for the intervention group. (PDF)

S6 File Leaflet for the control group. (PDF)

S7 File Post-intervention questionnaire, Q2. (PDF)
