# Supplementary material for: Effect of information about the benefits and harms of mammography on women’s decision making: The InforMa randomised controlled trial
Source: PLoS One. 2019 Mar 26;14(3):e0214057. doi: 10.1371/journal.pone.0214057 (PMC6435150; doi:10.1371/journal.pone.0214057)

CONVOCATORIA DE AYUDAS DE PROYECTOS DE INVESTIGACIÓN EN SALUD
MEMORIA DE SOLICITUD

Expediente Nº
PI14/00113

TITULO: **Participación de las mujeres en las decisiones y estrategias de detección precoz del cáncer de mama**

INVESTIGADOR/A PRINCIPAL: Montserrat Rué Monné

TIPO DE PROYECTO: ○ INDIVIDUAL ◉ COORDINADO ○ MULTICÉNTRICO

NOMBRE DEL IP COORDINADOR:
(Cumplimentar sólo en caso de proyectos coordinados)

DURACION: ☒ 3 AÑOS

RESUMEN (Objetivos y Metodología del Proyecto) **(Máximo 250 palabras)**

Los **objetivos** específicos de este subproyecto son: 1) Realizar una síntesis del conocimiento científico actual sobre toma de decisiones compartida (TDC) en la detección precoz del cáncer de mama (DPCM), y más concretamente sobre el desarrollo e implementación de herramientas de ayuda a la toma de decisiones (HATD); 2) Evaluar la efectividad de la HATD, frente a la atención habitual, sobre la elección informada de las mujeres, conflicto decisional, intención de participar en la DPCM, y satisfacción; 3) Evaluar las preferencias de las mujeres entre un programa de DPCM uniforme como el actual y un programa adaptado al riesgo individual; y 4) Realizar un análisis coste-efectividad y daño-beneficio de un programa de DPCM adaptado al riesgo individual en comparación a un programa de DPCM uniforme, en un contexto de TDC.

Metodología: 1) Revisión sistemática de la literatura; 2) Ensayo controlado aleatorizado (ECA) en 400 mujeres que van a ser invitadas por primera vez en programas de DPCM de Cataluña y Canarias; 3) Estudio cualitativo y encuesta sobre elicitación de preferencias; y 4) Análisis de coste-efectividad y daño-beneficio mediante ratios incrementales y frontera de Pareto.

TITLE: Women participation in decisions and strategies on early detection of breast cancer

ABSTRACT (Objectives and Methodology of the project)

The specific **objectives** of this subproject are: 1) Conduct a synthesis of current scientific knowledge on shared decision making (SDM) in the early detection of breast cancer (EDBC), and more specifically on the development and implementation of decision aids (DA); 2) Evaluate the effectiveness of DA versus usual care, on informed choice, decisional conflict, intention to participate in the EDBC program, and satisfaction; 3) Evaluate the preferences of women between the standard program of EDBC and a program adapted to individual risk; and 4) Conduct cost-effectiveness and harm-benefit analyses of risk-based EDBC compared to uniform EDBC, in a SDM context.

Methodology: 1) Systematic review of the literature; 2) Randomized controlled trial (RCT) in 400 women who will be invited for the first time in EDBC programs of Catalonia and the Canary Islands; 3) Qualitative study and survey of preference elicitation; and 4) Cost-effectiveness and harm-benefit analyses using the incremental ratios and the Pareto frontier.

Expediente Nº PI14/00113

INVESTIGADOR/A PRINCIPAL: Montserrat Rué Monné

MEMORIA DE SOLICITUD PROYECTO DE INVESTIGACIÓN EN SALUD
SECCIÓN ANTECEDENTES Y ESTADO ACTUAL DEL TEMA
Finalidad del proyecto, antecedentes y estado actual de los conocimientos científico-técnicos, grupos nacionales o internacionales que trabajan en la línea específica del proyecto o en líneas afines.
Citar las referencias en el apartado siguiente: Bibliografía más relevante. Máximo 3 páginas.

Los programas poblacionales de cribado de cáncer de mama (CM) se extendieron en España durante la década de 1990. En aquellos años había un consenso generalizado sobre el beneficio del cribado, derivado de los resultados de varios ensayos clínicos poblacionales en EEUU y el norte de Europa que estimaban una reducción estadísticamente significativa y clínicamente relevante de la mortalidad por cáncer de mama (Rutqvist, 1990).

La controvertida revisión sistemática de Gotzsche et al. publicada en the Lancet (Gotzsche, 2000) marcó el inicio de un debate, aún vigente, sobre los beneficios y efectos adversos del cribado. La recomendación de cribado bienal entre 50 y 74 años del US Preventive Services Task Force el año 2009 causó una intensa reacción en contra por parte de varias sociedades médicas y un sector de de la población (USPSTF, 2009). Por un lado, los avances en los tratamientos adyuvantes, el abordaje multidisciplinario del tratamiento de la enfermedad y la más rápida identificación de los síntomas por parte de la población probablemente han contribuido a reducir, en términos relativos, el beneficio del cribado. Por otro, los resultados de la literatura sobre efectos adversos -más concordantes al estimar el riesgo de falsos positivos y menos al estimar el sobrediagnóstico y sobretratamiento-, muestran que los potenciales efectos negativos no son insignificantes.

Más de dos décadas después de la introducción del cribado poblacional del CM, la evidencia sobre el balance beneficios-efectos adversos no es concluyente. Este hecho está conduciendo a un replanteamiento del cribado poblacional. Dos son las propuestas que están tomando fuerza. La primera, trata sobre la conveniencia y el deber de informar a la población de las posibles consecuencias del cribado, tanto de los beneficios como de los efectos adversos. Algunos proponen no dedicar más energía a aumentar la participación en el programa o a valorar qué grupos de edad deben integrar la población diana y dedicarla a informar a las mujeres para que tomen la mejor decisión teniendo en cuenta sus preferencias y valores (Stefanek, 2011, Strech 2014, Hersch 2014). La segunda propuesta supone la adaptación de la estrategia de cribado al riesgo individual de CM. Algunos estudios recientes (Vilaprinyó 2014, Schousboe, 2012; Ayer, 2012) basados en modelos matemáticos, sugieren que el cribado personalizado puede aumentar el número de vidas salvadas en mujeres de alto riesgo y al mismo tiempo disminuir las complicaciones innecesarias en mujeres de bajo riesgo. Ambas propuestas están tomando fuerza en varios países europeos con programas poblacionales de cribado.

La toma de decisiones compartida en la detección precoz del cáncer

El papel de los ciudadanos en las decisiones que afectan a su salud está cambiando. Factores individuales como la mejora del nivel educativo y el acceso a la información médica, y factores colectivos como la formación de grupos de pacientes y los cambios en la legislación (consentimiento informado, principio de autonomía) han motivado la transición de un modelo paternalista a un modelo de corresponsabilidad médico-paciente.

La toma de decisiones compartida (TDC) se ha propuesto en los últimos años como un modelo de atención sanitaria de interés para la implicación de pacientes en las decisiones sobre su tratamiento, especialmente en aquellas situaciones en las que existe incertidumbre y la evidencia científica informa de un balance entre beneficios y riesgos que resulta similar para dos o más opciones terapéuticas (Edwards 2009). La TDC implica un proceso de comunicación y negociación en el cual el profesional sanitario aporta conocimiento técnico sobre el problema de salud y las ventajas e inconvenientes de las intervenciones diagnósticas y terapéuticas disponibles, mientras que el paciente aporta el conocimiento sobre sus propios valores, preferencias y preocupaciones sobre las opciones mostradas (Ruiz-Azarola, 2012). Por tanto, se trata de un modelo donde el paciente mantiene una posición activa, compartiendo con el médico la responsabilidad de la decisión mediante un proceso interactivo de comunicación y negociación. Las herramientas de ayuda a la toma de decisiones compartida (HATD) en la detección precoz del cáncer son materiales informativos diseñados para ayudar a la toma de decisiones en salud incorporando los valores individuales y preferencias.

Aunque en los últimos 10 años la TDC y la atención centrada en el paciente han sido temas recurrentes en la literatura médica, todavía queda mucho qué hacer para aumentar el conocimiento y la participación de los pacientes en las decisiones que afectan a su salud. Un estudio reciente sobre decisiones en salud, en Canadá, muestra que la población participa menos en las decisiones sobre cribado que en decisiones terapéuticas y que la discusión sobre efectos adversos es prácticamente inexistente (Fowler, 2013). Los autores señalan el contraste entre esta falta de discusión y la creciente preocupación en la literatura médica sobre las desventajas de la mamografía en CM y del antígeno PSA en cáncer de próstata. Otro estudio muestra que en Alemania, Italia, España y Francia, los folletos informativos para las mujeres que participan en programas de cribado de CM han variado muy poco

en los últimos años y que no se proporciona suficiente información sobre efectos adversos (Gummersbach, 2010). Dos estudios evaluaron el impacto de materiales de ayuda a la decisión en programas poblacionales de cribado del CM (Webster 2007; Mathieu 2010). En ambos, las mujeres del grupo intervención mostraron más conocimientos sobre el cribado, más competencia decisional, y participaron menos en el programa. En Alemania actualmente se está realizando un estudio, aleatorizado y controlado, sobre el efecto de un folleto informativo que presenta abiertamente los potenciales beneficios y efectos adversos del cribado del CM (Gummersbach, 2013). En España ha habido diversas experiencias dirigidas a fomentar la participación activa de los pacientes en las decisiones que afectan su salud (Fernández de Sanmamed, 2007; Ruiz-Arazola, 2012; Izquierdo, 2010; Perestelo, 2013). Se presentan más detalles sobre la TDC y las HATD en el subproyecto coordinado.

La detección precoz adaptada al riesgo individual: un nuevo paradigma de la prevención del CM

La evidencia reciente sobre la relación beneficios-efectos adversos está originando propuestas de detección precoz adaptadas al riesgo individual. El Institute of Medicine de los EEUU el año 2005 ya identificó que el cribado personalizado era crucial para mejorar el diagnóstico precoz del cáncer de mama.

Existen diversos instrumentos diseñados para medir la probabilidad individual de desarrollar CM (Gail, 2011; Tyrer, 2004). Tales instrumentos fueron desarrollados con referencia a la población norteamericana y como criterio de elegibilidad para la quimioprevención primaria con tamoxifeno, extendiéndose después a otros contextos y usos. Su utilidad para diferenciar perfiles de riesgo en el contexto de la detección precoz ha sido cuestionada, por su baja capacidad de discriminación y precisión, no siendo recomendable su uso en su forma original (Burón, 2013; Arrospide 2013). En los últimos años se ha propuesto incorporar la densidad mamaria,para mejorar la precisión de la estimación del riesgo, considerando que actúa como variable de efecto biológico precoz. Las mamas más densas tienen más tejido glandular y conjuntivo, que tejido adiposo. Las mujeres con mamas densas tienen un riesgo entre 2 y 6 veces más alto de desarrollar un cáncer de mama (Boyd, 2010). Podría reflejar una exposición acumulada del estroma mamario a hormonas y factores del crecimiento celular. La densidad mamaria es un factor asociado al riesgo global de CM, independientemente de otros factores considerados en el cálculo (Tice, 2005). Se ha propuesto un modelo basado en el concepto “edad del tejido mamario”, más que en la edad cronológica de la mujer, para valorar la susceptibilidad de desarrollar CM (Pike, 2004). Por último, la densidad mamaria también se asocia con la dificultad en el diagnóstico mamográfico del cáncer y podría originar con mayor frecuencia un falso negativo en las mamografías de cribado. Algunos autores plantean incluir en los modelos predictivos de riesgo de desarrollar CM la susceptibilidad genética. Sin embargo, sólo una pequeña parte del riesgo relativo familiar se explicaría por las variantes genéticas descubiertas hasta la fecha.

A pesar de las limitaciones de los instrumentos de medida del riesgo individual, varios trabajos de modelización matemática han evaluado el coste-efectividad o riesgo-beneficio de estrategias de cribado adaptadas al riesgo. Schousboe et al. 2012 propusieron considerar cuatro factores de riesgo (edad, historia familiar, densidad mamaria y biopsias previas) para determinar la edad al inicio y la periodicidad de los exámenes de cribado. En base al análisis de coste-efectividad, recomendaron estrategias de cribado variables tales como examinar bienalmente a las mujeres de 40 a 50 años con alguno de los otros tres factores de riesgo, o cada 3-4 años a las mujeres de 50 a 79 años con baja densidad mamaria y sin otros factores de riesgo. Van Ravesteyn et al. (2012) estimaron que el cribado en mujeres de 40 a 49 años con riesgo de cáncer dos veces superior a la población general presenta un ratio daño-beneficio similar al del cribado en mujeres de 50 a 74 años. Sería interesante disponer de una estimación del riesgo de CM de las mujeres de 40 a 49 años que se someten a mamografías periódicas de cribado oportunista (65% de las mujeres de este grupo de edad según la encuesta de Salud de Cataluña del año 2012). Recientemente, Vilaprinyó et al. (2014) han mostrado que las estrategias de cribado basadas en el riesgo resultan en mayor beneficio que los programas de cribado uniformes. Su trabajo estima que un programa de cribado variable según el riesgo podría obtener un beneficio similar al de los programas actuales, reduciendo un 10% los costes y un 20% los resultados adversos (falsos positivos y sobrediagnóstico).

Experiencias o proyectos sobre cribado personalizado en otros países

Actualmente en Europa están en marcha varios proyectos que tienen por objetivo la detección precoz personalizada del CM. En el proyecto ASSURE: Adapting Breast Cancer Screening Strategy Using Personalised Risk Estimation, participan Holanda, Alemania, Bélgica, Reino Unido, Dinamarca y España. Se propone añadir a la mamografía habitual a) resonancia magnética para las mujeres de riesgo más elevado (5% del total), y b) ecografía en 3D para las de riesgo intermedio y/o mamas densas (35%). El estudio PROCAS en el área de Manchester (Reino Unido) tiene por objetivo predecir el riesgo de CM mediante información sobre los factores de riesgo más importantes: antecedentes familiares, estilo de vida, densidad mamaria y características genéticas (a partir de muestras de saliva). La medida del riesgo va acompañada de un asesoramiento personalizado sobre la periodicidad de las pruebas y también sobre tratamiento preventivo en mujeres de riesgo elevado. El estudio KARMA del Karolinska Institute tiene varios subproyectos con objetivos diversos, entre ellos: identificar las características de la densidad mamaria que predisponen al cáncer, identificar factores de riesgo genéticos, y predecir el riesgo individual de forma más precisa que mediante los modelos disponibles actualmente. El Tailored Breast Screening Trial (Eugenio Paci, Italia) utiliza la densidad mamaria para adaptar el intervalo entre mamografías. En los EEUU el programa PROSPR, del National Cancer Institute, EEUU, tiene como objetivo mejorar el cribado de CM, colon y cérvix. Para el CM los objetivos son evaluar la detección con tomosíntesis, y personalizar el tratamiento de los carcinomas ductales in situ a través de marcadores pronósticos. El estudio Breast Radiology Evaluation and Study of Tissues (BREAST) es un proyecto conjunto del National Cancer Institute y el consorcio Breast Cancer Surveillance, cuyo objetivo es estudiar

las características radiológicas, histológicas, moleculares y bioquímicas asociadas a la densidad mamaria para entender cómo la densidad mamaria promueve la transformación neoplásica del epitelio.

El presente proyecto aportará información útil tanto para el diseño de la HATD como para el de una estrategia personalizada.

Evaluación económica

Si se pretende asignar los recursos escasos entre programas alternativos de detección precoz de la forma más eficiente posible, es imprescindible realizar un análisis de evaluación económica. Respecto de las tecnologías sanitarias, la evaluación económica estudia el impacto de una nueva intervención sanitaria sobre el estado de salud de los usuarios, comparándolo con la variación de los costes al aplicar esta nueva tecnología. Destaca por su importancia el análisis de coste – efectividad, que mide los costes en unidades monetarias y los resultados en años de vida ajustados por calidad (AVACs).

El análisis de costes distingue entre costes sanitarios, costes no sanitarios y coste de transferencias (Puig-Junoy, 2001). Los costes sanitarios incluyen los costes directos de los bienes y servicios utilizados en el tratamiento y los costes futuros, normalmente asociados al problema de salud evaluado, pudiendo distinguir entre los generados durante los años de vida ganados y durante los años que el individuo habría vivido igualmente sin intervención. Los costes no sanitarios se identifican principalmente con la productividad laboral perdida derivada de la morbilidad y mortalidad. La productividad laboral perdida se ha medido tradicionalmente a través de la teoría del capital humano. A pesar de que este método ha sido criticado en multitud de ocasiones no hay consenso en otra forma de valoración alternativa. Contemplar únicamente los costos friccionales relativos al reemplazo del trabajador enfermo (Koopmanschap, 1995) o incorporar su cuantía en el valor de los AVACs (Gold, 1996; Siegel, 1996), ha recibido fuertes críticas y ha abierto un debate aún no cerrado.

El análisis de los costes no sanitarios también puede recoger el impacto, presente y futuro, del tratamiento en los presupuestos públicos (consumo de servicios sociales) así como costes monetarios (transporte) o de tiempo (tiempos de espera) soportados por el paciente y sus acompañantes. Los costes de transferencia son los producidos por los flujos monetarios entre individuos o bien desde el sector público, sin consumo de recursos; raramente se incluyen en el análisis de evaluación económica, sobre todo si se hace desde el punto de vista social, puesto que en ese caso son cero.

FINALIDAD DEL PROYECTO

Este estudio se enmarca en el debate sobre beneficios y efectos adversos del cribado de CM, en el que la necesidad de informar más y mejor a la población y de compartir con ella las decisiones sobre su salud no sólo queda patente sino que es recomendación implícita del conjunto de la comunidad científica y social. Además, se sitúa en un momento de reflexión sobre las estrategias a seguir, en el que cada vez toma más fuerza la idea de cribar según el riesgo individual, precisamente para optimizar el balance riesgo-beneficio. Y por supuesto, en un contexto general de contención del gasto, en el que todas y cada una de las acciones sanitarias no sólo han de ser evaluadas en términos de impacto en salud, sino en términos económicos.

Expediente Nº PI14/00113

INVESTIGADOR/A PRINCIPAL: Montserrat Rué Monné

MEMORIA DE SOLICITUD PROYECTO DE INVESTIGACIÓN EN SALUD
SECCIÓN ANTECEDENTES Y ESTADO ACTUAL DEL TEMA

Citar las referencias incluidas en el apartado anterior: Antecedentes y Estado actual. **Máximo 1 página.**

- Arrospide A, Forné C, Rué M, et al. An assessment of existing models for individualized breast cancer risk estimation in a screening program in Spain. BMC Cancer 2013;13:587.
- Ayer T, Alagoz O, Stout NK. A POMDP Approach to Personalize Mammography Screening Decisions. Operations Research 2012, 60:1019-1034.
- Boyd NF, Martin LJ, Bronskill M Y, et al. Breast tissue composition and susceptibility to breast cancer. J Natl Cancer Inst 2010;102:1224-37.
- Burón A, Vernet M, Román M, et al. Can the Gail model increase the predictive value of a positive mammogram in a European population screening setting? Results from a Spanish cohort. Breast 2013;22:83-8.
- Costa-Font J. Participación colectiva y revelación de preferencias sobre programas sanitarios: un enfoque de sistema sanitario. Gac Sanit 2005; 19: 242-52.
- Dormandy E, Michie S, Hooper R, et al. Informed choice in antenatal Down syndrome screening: a cluster-randomised trial. Patient Educ Couns 2006; 61: 56-64.
- Edwards A, Elwyn G. Shared Decision-Making in Health Care: Achieving evidence-based patient choice. Second edition. New York: Oxford University press, 2009.
- Fernández de Sanmamed MJ, Ballester Torrens M, et al. Comprensión de un documento que informa a los ciudadanos sobre los beneficios y los riesgos del cribado para el cáncer de próstata. Estudio mediante entrevistas semiestructuradas. Rev Esp Salud Publica 2007;81:289-305.
- Fowler FJ, Gerstein BS, Barry MJ. How patient centered are medical decisions? Results of a national survey. JAMA Intern Med. 2013;27:1-7.
- Gail MH. Personalized estimates of breast cancer risk in clinical practice and public health. Stat Med 2011;30:1090-1104.
- Gold MR, Siegel JE, Russell LB, et al. Cost–effectiveness in health and medicine. 1996; New York: Oxford University Press.
- Gotzsche PC, Olsen O. Is screening for breast cancer with mammography justifiable? Lancet 2000 Jan 8;355:129-34.
- Gummersbach E, Piccoliori G, Zerbe CO, et al. Are women getting relevant information about mammography screening for an informed consent: A critical appraisal of information brochures used for screening invitation in Germany, Italy, Spain and France. Eur J Public Health 2010;20:409-14.
- Gummersbach E, Schmitten J, Abholz HH, et al. Effects of different information brochures on women's decision-making regarding mammography screening: study protocol for a randomized controlled questionnaire study. Trials 2013, 14:319.
- Hersch J, Barratt A, Jansen J, et al. The effect of information about overdetection of breast cancer on women's decision-making about mammography screening: study protocol for a randomised controlled trial. BMJ Open 2014; 15;4:e004990.
- Izquierdo F, Blasco JA. Herramienta de Ayuda para la Toma de Decisiones compartida en cáncer de mama. Madrid: Plan de Calidad para el SNS del MSPSI. Unidad de Evaluación de Tecnologías Sanitarias, Agencia Laín Entralgo; 2010.
- Koopmanschap MA, Rutten FFH, Van Ineveld BM, et al. The friction cost method for measuring indirect costs of disease. J Health Econ 1995; 14: 171-189.
- Mathieu E, Barratt AL, McGeechan K, et al. Helping women make choices about mammography screening: an online randomized trial of a decision aid for 40-year-old women. Patient Educ Couns 2010, 81:63.
- O'Connor AM. Validation of a decisional conflict scale. Med Decis Making 1995; 15: 25-30.
- Perestelo-Perez L, Perez-Ramos J, Abt-Sacks A, et al. Promoción de la participación ciudadana en cuidados de salud a través de PyDEsalud.com. Gac Sanit 2013 Mar 1.
- Pike MC, Pearce CL, Wu AH. Prevention of cancers of the breast, endometrium and ovary. Oncogene 2004;23:6379-6391.
- Puig-Junoy J, Ortún V, Pinto JL. Los costes en la evaluación económica de tecnologías sanitarias. Atención Primaria 2001; 3:186-189.
- Ruiz-Azarola A, Perestelo-Pérez L. Participación ciudadana en salud: formación y toma de decisiones compartida.
Informe SESPAS 2012. Gac Sanit 2012;26 Suppl 1:158-61.
- Rutqvist LE, Miller AB, Andersson I, et al. Reduced breast-cancer mortality with mammography screening--an assessment of currently available data. Int J Cancer Suppl 1990;5:76-84.
- Schousboe JT, Kerlikowske K, Loh A, Cummings SR. Personalizing mammography by breast density and other risk factors for breast cancer: Analysis of health benefits and cost-effectiveness. Ann Intern Med 2011;155:10-20.
- Siegel JE, Weinstein MC, Russell LB, et al. Recommendations for reporting cost-effectiveness analyses. Panel on Cost-Effectiveness in Health and Medicine. JAMA 1996; 276: 1339–1341.
- Stefanek ME. Uninformed compliance or informed choice? A needed shift in our approach to cancer screening. J Natl Cancer Inst 2011;103:1821-6.
- Strech D. Participation rate or informed choice? Rethinking the European key performance indicators for mammography screening. Health Policy 2014; 115: 100-103.
- Tice JA, Cummings SR, Ziv E, et al. Mammographic breast density and the Gail model for breast cancer risk prediction in a screening population. Breast Cancer Res Treat 2005;94:115-22.
- Tyrer J, Duffy SW, Cuzick J. A breast cancer prediction model incorporating familial and personal risk factors. Stat Med 2004;23:1111-30.
- US Preventive Services Task Force. Screening for breast cancer: U.S. Preventive Services Task Force recommendation statement. Ann Intern Med. 2009;151:716,26, W-236.
- van Ravesteyn NT, Miglioretti DL, Stout NK, et al. Tipping the balance of benefits and harms to favor screening mammography starting at age 40 years: A comparative modeling study of risk. Ann Intern Med 2012;156:609-17.
- Webster P, Austoker J. Does the English breast screening program's information leaflet improve women's knowledge about mammography screening? A before and after questionnaire survey. J Public Health 2007, 29:173–177.
- Vilaprinyó E, Forné C, Carles M, et al. Cost-effectiveness and harm-benefit analyses of risk-based screening strategies for breast cancer. Plos One 2014; 9:e86858.

Expediente Nº PI14/00113

INVESTIGADOR/A PRINCIPAL: Montserrat Rué Monné

MEMORIA DE SOLICITUD PROYECTO DE INVESTIGACIÓN EN SALUD
SECCIÓN HIPÓTESIS Y OBJETIVOS

(Ajústese al espacio disponible)

HIPÓTESIS

H1. La integración de la mejor evidencia disponible sobre daño-beneficio del cribado del cáncer de mama (CM), las preferencias de las mujeres, y la comunicación adaptada al contexto social, cultural y asistencial, mejora la toma de decisiones informada, reduce el conflicto decisional y tiene una elevada aceptabilidad en la detección precoz del cáncer de mama (DPCM).

H2. Las mujeres valoran positivamente la posible implantación de un programa de DPCM (PDPCM) adaptado al riesgo individual. Su intención de participar en él, en un contexto de toma de decisiones compartida (TDC), sería elevada (más del 50%).

H3. Un PDPCM adaptado al riesgo individual, en un contexto de TDC, contribuiría a una mayor eficiencia del sistema sanitario y a una mejora de la relación daño-beneficio del cribado.

OBJETIVOS

Globales de este subproyecto

1) Evaluar el impacto de una herramienta de ayuda a la toma de decisiones (HATD) sobre el proceso de toma de decisión y la participación en un PDPCM.

2) Evaluar las preferencias de las mujeres en relación a un hipotético PDPCM adaptado al riesgo y evaluar su eficiencia y relación daño-beneficio respecto a un programa de cribado uniforme.

Específicos de este subproyecto

1. Realizar una búsqueda y síntesis del conocimiento científico actual sobre TDC en la DPCM, y más concretamente sobre el desarrollo e implementación de HATD en dicha área.

2. Evaluar la efectividad de la HATD, frente a la atención habitual, sobre la elección informada de las mujeres y otras medidas de resultado (conflicto decisional, intención de participar en el PDPCM, satisfacción).

3. Evaluar las preferencias de las mujeres entre dos programas alternativos: un PDPCM uniforme como el actual y un PDPCM adaptado al riesgo individual, ambos en un contexto de TDC.

4. Realizar un análisis coste-efectividad y daño-beneficio de un PDPCM adaptado al riesgo individual en comparación a un PDPCM uniforme, ambos en un contexto de TDC.

Expediente Nº PI14/00113

INVESTIGADOR/A PRINCIPAL: Montserrat Rué Monné

MEMORIA DE SOLICITUD PROYECTO DE INVESTIGACIÓN EN SALUD
SECCIÓN PROYECTOS COORDINADOS

En caso de Proyectos Coordinados, el COORDINADOR deberá indicar:
- Objetivos globales del proyecto coordinado, la necesidad de dicha coordinación y el valor añadido que se espera obtener de la misma.
- Objetivos específicos de cada subproyecto (deben están recogidos además en la memoria de cada subproyecto)
- Interacción entre los distintos objetivos, actividades y subproyectos.
- Los mecanismos de coordinación previstos para la eficaz ejecución del proyecto. Máximo 3 páginas.

Objetivos globales del proyecto coordinado

1) Diseñar, desarrollar y evaluar una herramienta de ayuda a la toma de decisiones (HATD) para facilitar la toma de decisiones compartida (TDC) sobre detección precoz del cáncer de mama (DPCM).

2) Evaluar las preferencias de las mujeres en relación a un hipotético programa de DPCM adaptado al riesgo y evaluar su eficiencia y relación daño-beneficio respecto a un programa de cribado uniforme.

Específicos, subproyecto Canarias (Subproyecto 1)

1. Realizar una búsqueda y síntesis del conocimiento científico actual sobre TDC en la DPCM, y más concretamente sobre el desarrollo e implementación de HATD en dicha área.

2. Identificar creencias, conocimientos y necesidades de información de las mujeres respecto a la DPCM.

3. Explorar las actitudes, preferencias, barreras y facilitadores percibidos por las mujeres y profesionales sanitarios respecto a la incorporación del modelo de TDC en la DPCM, así como sus actitudes y aceptabilidad de un modelo de cribado basado en el riesgo individual, en contraposición al modelo actual (bienal a partir de los 50 años).

4. Elaborar una HATD sobre la DPCM, desarrollada de forma colaborativa con la población diana y los profesionales sanitarios (en consonancia con los resultados obtenidos en los objetivos 1 a 3) y evaluar su aceptabilidad por parte de la mujeres y profesionales directamente implicados en la DPCM.

5. Evaluar la efectividad de la HATD, frente a la atención habitual, sobre la elección informada de las mujeres y otras medidas de resultado (conflicto decisional, intención de participar en la DPCM, satisfacción).

Específicos, subproyecto Cataluña (Subproyecto 2)

1. Realizar una búsqueda y síntesis del conocimiento científico actual sobre TDC en la DPCM, y más concretamente sobre el desarrollo e implementación de HATD en dicha área.

2. Evaluar la efectividad de la HATD, frente a la atención habitual, sobre la elección informada de las mujeres y otras medidas de resultado (conflicto decisional, intención de participar en el PDPCM, satisfacción).

3. Evaluar las preferencias de las mujeres entre dos programas alternativos: un PDPCM uniforme como el actual y un PDPCM adaptado al riesgo individual, ambos en un contexto de TDC.

4. Realizar un análisis coste-efectividad y daño-beneficio de un PDPCM adaptado al riesgo individual en comparación a un PDPCM uniforme, ambos en un contexto de TDC.

Interacción de los distintos objetivos, actividades y subproyectos

Este proyecto está basado en el análisis y posterior desarrollo de dos inquietudes que actualmente existen en el

contexto de los PDPCM: programas adaptados al riesgo individual y necesidad de TDC.

El actual proyecto coordinado se presenta mediante dos subproyectos, con IP. Amado Javier Rivero en Canarias (Subproyecto 1) y con IP Montserrat Rué en Cataluña (Subproyecto 2). Los dos objetivos globales equivalen a la especificación de las dos inquietudes que se han esquematizado en el apartado Antecedentes de la memoria de ambos grupos. La HATD se va a desarrollar conjuntamente en los dos subproyectos. El equipo investigador del subproyecto 1 tiene amplia experiencia en el desarrollo de HATD en el ámbito de la salud y actualmente está desarrollando una HATD para la detección precoz del cáncer de colon. El equipo investigador del subproyecto 2 va a aportar la experiencia y conocimiento de varios años de trabajo en evaluación de la detección precoz del cáncer de mama. La colaboración de los dos equipos es necesaria para asegurar el éxito del proyecto.

Inicialmente, los investigadores de ambos subproyectos revisarán la literatura correspondiente a estos dos objetivos globales y tendrán en cuenta el actual estado de desarrollo de ambos temas, tanto en España como en otros países occidentales (Objetivo específico 1 de ambos subproyectos). Las conclusiones que se extraigan, se pondrán en común, se debatirán y las conclusiones servirán para iniciar el trabajo propio de esta propuesta.

El punto de partida de los restantes objetivos específicos es la toma de decisiones compartidas. Por este motivo, los objetivos 2 y 3 del subproyecto 1 se proponen identificar conocimiento y necesidades de información de las mujeres y explorar las actitudes y preferencias de mujeres y profesionales respecto a la oportunidad de incorporar el modelo de TDC en el actual programa de cribado. En base a los resultados de estos dos objetivos, el subproyecto 1 coordinará las tareas y desarrollará la HATD oportuna.

La HATD será la base del desarrollo del resto de objetivos. En primer lugar, debe evaluarse la efectividad la HATD (Objetivo específico 2 del subproyecto 2 y 5 del subproyecto 1). Se propone un estudio experimental, controlado y aleatorizado en dos etapas. Como puede comprobarse en el apartado metodología de las memorias de este proyecto, las mujeres invitadas a participar provienen tanto del entorno de Cataluña (urbano y rural) como de Canarias. Por este motivo se elabora y desarrolla conjuntamente. Pero, mientras la recogida de datos puede hacerse en ambas localizaciones, el diseño, coordinación y análisis estadístico de los resultados corresponde al subproyecto 2. Los resultados deben dar respuesta a las hipótesis planteadas. Concretamente H1 y H4 del subproyecto 1 y H1 del subproyecto 2 están directamente relacionadas con este objetivo.

Del mismo modo, utilizando la HATD se pretende conocer las preferencias de las mujeres (Objetivos específicos 3 de los dos subproyectos) y la valoración y aceptabilidad de los profesionales (Objetivo 3 del subproyecto 1) sobre la posibilidad de implantar un programa de cribado adaptado al riesgo individual . El desarrollo del objetivo se plantea mediante encuesta a tres grupos de mujeres en dos fases. La primera de ellas, que tiene carácter cualitativo, se realiza en el subproyecto 1 y la segunda, de carácter cuantitativo, en el subproyecto 2.

Finalmente, se propone realizar un análisis coste-efectividad y daño-beneficio de un programa adaptado al riesgo individual en comparación con el programa actual, pero en un contexto de TDC y en base al resultado del apartado anterior para el que suponemos que un porcentaje elevado de mujeres estarán dispuestas a participar en un programa distinto del actual (H2 del subproyecto 2 y H3 del subproyecto 1). El subproyecto 2 coordinarà este apartado.

Finalmente, ambos grupos de investigación colaboraran estrechamente en la discusión de las conclusiones de todos los apartados y redacción de los artículos e informes correspondientes.

Mecanismos de coordinación previstos para la eficaz ejecución del proyecto

1. El proyecto dispondrá de un Comité de Dirección (CD) integrado por dos investigadores de cada subproyecto y dirigido por Montserrat Rué como investigadora coordinadora. Este CD llevará a cabo su actividad habitual de coordinación por teleconferencias, uso compartido de herramientas colaborativas de gestión automatizada del proyecto, así como de reuniones presenciales (una al año).

2. Las bases de datos específicamente desarrolladas para este proyecto estarán diseñadas en plataforma web para su uso integrado a la vez que autónomo entre los dos subproyectos.

3. La coordinación prevé la difusión de los resultados, aplicándose para ello la política de publicaciones colaborativas desarrollada en el marco de REDISSEC. Se buscará por un lado la transferencia de los resultados en el marco de la ECSNS y de los PDPCM de las CCAA, por otro se quiere obtener publicaciones de alto impacto científico, aspecto

éste avalado por la propia pertenencia a REDISSEC.

Cronograma común a los dos subproyectos

Año 2015. Semestre 1
Común (C). Reunión presencial para la puesta en común del objetivo O1 de ambos subproyectos. Comunicación electrónica mensual para el resto de objetivos.
Subproyecto 1 (S1). Obj. 1: Realización del protocolo. Búsqueda y selección de artículos. Análisis y síntesis de resultados. Obj. 2 y 3: Desarrollo protocolo.
Subproyecto 2 (S2). Obj. 1: Revisión de la literatura. Análisis y síntesis de resultados.

Año 2015. Semestre 2
C. Comunicación electrónica mensual para seguimiento del proyecto.
S1. Obj. 2 y 3: Reclutamiento para grupos focales, transcripción y análisis de datos, reclutamiento para grupos consultivos.
S2. Obj. 4: Análisis de costes.

Año 2016. Trimestres 1, 2 y 3
C-. Reunión presencial para discutir los resultados de los objetivos Obj. 2 y 3 del S1 y de los resultados de la prueba piloto del S2 (Obj. 2). Comunicación electrónica mensual para el resto de objetivos.
S1. Obj. 4: Desarrollo de la HATD y testado de campo.
S2. Obj. 3: Preparación del estudio de preferencias. Obj. 2: Prueba piloto Ensayo Controlado Aleatorizado (ECA)

Año 2016. Trimestre 4
C. Comunicación electrónica del desarrollo de la ECA.
S1. Obj. 5: ECA-muestra correspondiente a Canarias.
S2. Obj. 2: ECA-muestra correspondiente a Cataluña. Estudio de preferencias.

Año 2017. Trimestre 1 y 2
C. Comunicación electrónica del desarrollo de la ECA.
S1 y S2. Obj. 2: ECA.

Año 2017. Trimestre 3
C. Reunión presencial para debatir los resultados del estudio Coste-Efectividad y de los resultados del Obj. 3 de S2.
S2. Obj. 3: Estudio de preferencias. Análisis de datos. Obj. 4: Estudio de coste-efectividad

Año 2017. Trimestre 4
C. Comunicación electrónica.
S1 y S2. Elaboración informe final.

Expediente Nº PI14/00113

INVESTIGADOR/A PRINCIPAL: Montserrat Rué Monné

MEMORIA DE SOLICITUD PROYECTO DE INVESTIGACIÓN EN SALUD
SECCIÓN METODOLOGÍA
Diseño, sujetos de estudio, variables, recogida y análisis de datos y limitaciones del estudio. Máximo 3 páginas.

Objetivo 1: Realizar una búsqueda y síntesis del conocimiento científico actual sobre TDC en la DPCM, y más concretamente sobre el desarrollo e implementación de HATD en dicha área.

Este objetivo estará coordinado por el equipo investigador del subproyecto "Herramienta de Ayuda para la Toma de Decisiones (HATD) sobre la detección precoz del cáncer de mama" (IP: Amado Rivero), y es detallado en la memoria correspondiente de dicho subproyecto.

Objetivo 2. Evaluar la efectividad de HATD, frente a la atención habitual, sobre la elección informada de las mujeres y otras medidas de resultado (conflicto decisional, intención de participar en la DPCM, satisfacción).

Diseño: Estudio experimental aleatorizado en dos etapas y controlado. La figura 1 en el anexo resume el diseño del estudio.
Población de estudio: Mujeres de 49-50 años que en 2-4 meses van a ser invitadas a participar por primera vez en los programas de detección precoz del cáncer de mama (PDPCM) de Parc de Salut Mar (Barcelona), ICO-Hospitalet (Hospitalet de Llobregat), Regió Sanitària Lleida, y Canarias. Criterios de exclusión: 1) historia previa de cáncer de mama y/o mastectomía bilateral, 2) dificultad de comprensión idiomática, 3) problemas cognitivos para completar o entender los materiales.
Periodo de estudio: El periodo de inclusión comprenderá el cuarto trimestre de 2016 y el primer semestre de 2017. Se seguirán las mujeres hasta la cita del PDPCM.

Prueba piloto: Antes de iniciar el estudio experimental se realizará una prueba piloto con 10 mujeres de cada PDPCM participante para verificar la idoneidad de los procesos de reclutamiento y recogida de datos. Se realizará durante el tercer trimestre de 2016.

Selección de las mujeres y asignación a los dos grupos de estudio: Con la finalidad de reducir la contaminación intragrupal, la primera etapa del muestreo asignará aleatoriamente 10 Áreas Básicas de Salud (ABS) o conglomerados de los 4 PDPCM participantes a los grupos control (GC) e intervención (GI). Para maximizar la comparabilidad de los grupos GC y GI, se clasificarán las ABS de cada PDPCM según sus características socioeconómicas y se asignarán aleatoriamente por bloques de tamaño 2 a los grupos control (GC) e intervención (GI). Las fuentes de información serán las que utilizan los PDPCM para invitar a la población diana: Registro Central de Asegurados (RCA) en Cataluña y Registro de la Tarjeta Sanitaria en Canarias. En la segunda etapa del muestreo, se seleccionarán aleatoriamente 210 mujeres en cada programa de cribado (840 mujeres en total (ver apartado "Tamaño muestral")) entre las que cumplan el criterio de inclusión, distribuidas de manera uniforme entre las ABS del GI y GC. A todas las mujeres seleccionadas se les enviará por correo una carta de presentación del estudio y un sobre pre-franqueado con aceptación de participar en el estudio. Se realizará un seguimiento telefónico en el intervalo de dos semanas posteriores al envío para comprobar que han recibido la invitación y evaluar los criterios de inclusión/exclusión.

Recogida de datos antes de la intervención: A las mujeres que acepten participar se les enviará por correo el folleto informativo estándar sobre detección precoz propio de cada PDPCM, el cuestionario pre-intervención y un documento de consentimiento informado. Una semana más tarde, se contactarán por telefóno para obtener el consentimiento de forma verbal y realizar la encuesta pre-intervención. El cuestionario incluirá variables sociodemográficas, experiencia previa de cribado, factores de riesgo del CM, nivel de alfabetización de términos médicos, y uso de tecnologías de información y comunicación.

Intervención: Las mujeres del GI recibirán por correo la HATD en formato papel y la dirección que contiene la HATD en formato web. Se les facilitará asimismo un teléfono de contacto y un e-mail para ampliar la información y resolver sus dudas. Si lo desean, podrán concertar una cita presencial con un profesional sanitario del equipo investigador para reforzar el proceso de TDC. No se contempla la realización de una entrevista individual por un profesional sanitario a todas las mujeres porque los recursos de los PDPCM son limitados y el proyecto pretende valorar un escenario realista. No se realizará ninguna intervención en las mujeres del GC.

Recogida de datos después de la intervención:
Los dos grupos de estudio serán encuestados telefónicamente de nuevo al finalizar el periodo intervención. Las mujeres del GI se entrevistarán en el intervalo de 2-4 semanas después de haber enviado la HATD y las del GC en el intervalo de 2-4 semanas después de la entrevista pre-intervención. Se utilizará un cuestionario que incluirá conocimiento de aspectos relacionados con el cáncer de mama y la detección precoz; actitud hacia el cribado; opinión y preferencias respecto al grado de participación en la toma de decisiones; intención de participar en el programa de cribado; conflicto de decisión; satisfacción con la decisión; y grado de aceptación/utilidad de la HATD (en el GI). Se utilizarán escalas validadas cuya idoneidad ha sido demostrada en estudios previos (Hersch 2014, Mathieu 2010). Se monitorizarán las solicitudes de información adicional, ya sea telefónica y/o presencial, y también las entradas y tiempo de conexión a la HATD en web. Una vez transcurrida la visita correspondiente a la primera mamografía, se obtendrá la participación en los PDPCM y los motivos de no participación, si procede, de todas las mujeres

incluidas en el estudio.

Variable principal: Se considerará como **variable principal** de resultado la **elección informada** (Hersch 2014), de naturaleza dicotómica. Siguiendo el trabajo de Hersch et al., esta variable se obtendrá a partir de tres componentes: conocimientos, actitudes e intenciones. Una elección informada se caracteriza por la intersección de conocimiento adecuado y actitudes e intenciones consistentes. Cada una de las tres componentes se medirá mediante una escala que se convertirá en dicotómica mediante un punto de corte fijado a priori. Un conocimiento adecuado debe indicar comprensión básica de conceptos como falsos positivos, reducción de mortalidad y sobrediagnóstico. Las actitudes se medirán mediante una escala de seis items (Dormandy 2006). La intención de participar o no en el cribado se medirá mediante una escala de Likert con 5 categorías (dicotomizada 1-3: no, 4,5: sí). Las tres componentes de la variable principal también se analizarán separadamente.

Variables secundarias: Participación en el cribado, conflicto decisional (medida con la escala *Decisional Conflict Scale* (versión de 10 ítems adaptada para el estudio, O'Connor 1995), satisfacción con la decisión, y grado de aceptación/utilidad de la HATD en el GI.

Tamaño muestral: El análisis principal comparará la proporción de mujeres que toman una decisión informada en los dos grupos de estudio mediante la prueba ji-cuadrado y el intervalo de confianza (95%) de la diferencia de proporciones. Consideramos relevante una diferencia absoluta de 20%. Suponiendo que una de las proporciones del grupo es 50% (escenario más conservador) y estimando un coeficiente de correlación intraclase igual a 0.1 (muestreo por conglomerados), a fin de alcanzar una potencia superior a 80% para detectar una diferencia entre los grupos de 20% con un nivel de significación bilateral del 5%, se requieren 200 mujeres por grupo, 400 en total, 100 por programa participante. Este tamaño de muestra es suficiente para detectar una diferencia de 20% en las intenciones y una diferencia media menor que 0.35 desviaciones estándar en las escalas de conocimientos y actitudes (en base a los resultados de la literatura). Asumiendo que un 60% de las mujeres invitadas aceptan participar y que se producen un 20% de pérdidas de seguimiento, se invitarán a participar en el estudio 210 mujeres en cada uno de los PDPCM, 840 mujeres en total.

Análisis estadístico: Análisis comparativo de las variables principal y secundarias en los dos grupos de estudio mediante intervalos de confianza y pruebas de hipótesis ji-cuadrado (variables cualitativas), t de Student y U de Mann-Whitney (variables cuantitativas). Se utilizarán modelos de regresión múltiple lineal y logística para valorar la asociación entre las características de la mujer y las variables de resultado. Si la presencia de valores faltantes redujera en más de un 10% el número de registros de los modelos multivariables, se utilizaría un método de imputación múltiple y análisis de sensibilidad.

Objetivo 3: Evaluar las preferencias de las mujeres entre dos programas alternativos: un PDPCM uniforme como el actual y un PDPCM adaptado al riesgo individual, ambos en un contexto de TDC

Se realizará en dos fases: 1) GF de mujeres de la población diana del PDPCM (50-69 años), mujeres de 40 a 45 años, y profesionales sanitarios; 2) Encuesta a tres grupos de mujeres: 40-45 años, 50-55 años participantes en el PDPCM, y 50-55 años no participantes en el PDPCM. En la primera fase, desarrollada en el subproyecto coordinado, se explorarán las actitudes y la aceptabilidad de un modelo de cribado basado en el riesgo individual en contraposición al modelo actual (bienal a partir de los 50 años), y en la segunda, desarrollada en este subproyecto, se evaluarán las preferencias de las mujeres sobre el tipo de cribado, mediante una encuesta en profundidad y la intención de participar en un hipotético programa de riesgo individual mediante un ranking de preferencias. La inclusión de mujeres de 40 a 45 años se justifica porque los modelos basados en el riesgo individual contemplan incorporar a las mujeres para una primera evaluación del riesgo a partir de esa edad (Schousboe, 2012). Se plantearán distintos escenarios hipotéticos de cribado, con estimación de beneficios y efectos adversos del cribado, y se pedirá a las mujeres que expresen sus preferencias. Se adoptará la «perspectiva personal socialmente incluyente» (socially inclusive personal perspective) situada entre la perspectiva individual y la social en la que el individuo valora los programas colectivos entre los que él mismo es un potencial beneficiario (Costa-Font, 2004).

El contenido de la encuesta se fundamentará en la literatura así como en los resultados de la fase cualitativa desarrollada en el subproyecto coordinado, y se llevará a cabo telefónicamente, previo envío postal de la información del estudio, material informativo sobre los programas basados en el riesgo individual y obtención de su consentimiento a participar (en la carta se incluirá por escrito, pero se obtendrá de forma verbal durante la llamada). La muestra será aleatoria y estratificada según los tres grupos de estudio. Las mujeres de 50-55 años participantes en el PDPCM serán invitadas a participar en la encuesta una vez concluido el episodio de cribado por dos motivos: evitar interferencias entre el estudio y la participación en el Programa; y recoger mejor su opinión sobre el cribado basado en el riesgo individual, al permitir la comparación con la reciente experiencia en el cribado actual. Las fuentes de información de las mujeres de 40-45 años serán el RCA (Cataluña) y el Registro de la Tarjeta Sanitaria en Canarias, y de las mujeres de 50-55 años (participantes o no en el PDPCM) las bases de datos de los programas. Dado que el objetivo es recoger la variabilidad de respuestas según los perfiles sin necesidad de analizar comparativamente los grupos, la muestra se decidirá en base al estimador principal de aceptabilidad e intención de participar, calculando a priori un tamaño mínimo necesario de 120 mujeres, 40 por grupo de edad (aceptando un error alfa de 0.05, una precisión de +/- 0.1 unidades en un contraste bilateral para una proporción estimada de 0.5, la más conservadora, y un 20% de pérdidas de seguimiento).

Análisis estadístico: Se realizará un análisis descriptivo de las preferencias. Se estimará la proporción de mujeres que prefieren la detección precoz basada en el riesgo y su intervalo de confianza (95%), por estratos y global, teniendo en cuenta los pesos de

cada estrato en la población muestreada.

Objetivo 4: Realizar un análisis coste-efectividad y daño-beneficio de un PDPCM adaptado al riesgo individual en comparación a un PDPCM uniforme, ambos en un contexto de TDC.

En base al trabajo de Vilaprinyó et al. 2014 se elaborará un modelo probabilístico de coste-efectividad y daño-beneficio. Para cada alternativa se obtendrá el beneficio (reducción de mortalidad, AVACs), los daños (falsos positivos y sobrediagnóstico) y los costes, con un horizonte temporal a largo plazo y adoptando una perspectiva social.

Evaluación de los costes
Se detallan a continuación los costes que serán considerados y como serán identificados, medidos y valorados.

(1) Costes sanitarios directos (Vilaprinyó et al, 2014):
a) *Costes de detección:* personal inherente al programa, material fungible y depreciación del inmovilizado material, pruebas de cribado y pruebas complementarias. La información la facilitarán los PDPCM participantes.
c) Costes de información: costes fijos necesarios para facilitar la información (personal, infraestructuras) y HATD.
b) *Costes de tratamiento*. Se subdividirá el estudio en tres etapas: tratamiento inicial, seguimiento y costes de la enfermedad avanzada. Se consideran las siguientes categorías de costes: ingresos hospitalarios, visitas ambulatorias, quimioterapia, radioterapia, hormonoterapia y otros costes hospitalarios. Los costes de tratamiento se obtendrán del Hospital del Mar de Barcelona

(2) Costes no sanitarios:
a) Costes directos de tiempo y transporte de pacientes y acompañantes: salario medio bruto anual en función de la edad media de la población de estudio (INE/IDESCAT). Costes de transporte por Km publicados por la Administración Pública.
b) Costes indirectos de productividad: se estimarán en base a la pérdida o restricción de capacidad para trabajar valorada según el salario medio anual (INE).

(3) No se incluyen en el estudio los costes futuros ni los costes de transferencia.

Evaluación de la efectividad
Se obtendrán los AVAC de las dos alternativas analizadas en base a los resultados del modelo probabilístico y de los objetivos 1 y 2.1 del proyecto, que proporcionarán información sobre la distribución de factores de riesgo (excepto densidad mamaria), la predisposición hacia el cribado y la voluntad de participar en las dos alternativas de cribado.

Elaboración de los ratios incrementales coste – efectividad (ICER) y daño-beneficio
El proceso de TDC afecta tanto a los costes como a los resultados. Los costes varían como consecuencia de la elaboración de materiales y la transmisión de la información, así como por la decisión de algunas mujeres de participar o no en los programas alternativos y consecuentemente en el número de pruebas complementarias de diagnóstico y de tratamiento. La efectividad se verá afectada igualmente por esa decisión

El resultado del análisis coste-efectividad se especifica como un ratio incremental entre la diferencia de costes, medidos en euros y la variación de la efectividad medida en AVACs. Se expondrán por separado los resultados de la perspectiva privada, analizada desde el punto de vista del financiador, para que puedan compararse con otros que hayan utilizado esta perspectiva de análisis. De acuerdo con la bibliografía, se descontarán tanto los costes como los resultados a las tasas aconsejadas por las distintas Agencias de Evaluación (3%) y se realizará un análisis de sensibilidad de los principales parámetros.

Aspectos éticos y limitaciones (Ver anexo)

Expediente Nº PI14/00113

INVESTIGADOR/A PRINCIPAL: Montserrat Rué Monné

MEMORIA DE SOLICITUD PROYECTO DE INVESTIGACIÓN EN SALUD
SECCIÓN PLAN DE TRABAJO

Etapas de desarrollo y distribución de las tareas de todo el equipo investigador, y las asignaciones previstas para el personal técnico que se solicita. Indicar además el lugar/centro de realización del proyecto.
(Ajústese al espacio disponible)

Etapas del desarrollo

Primer año: En los dos primeros meses (mes 1 y 2), se desarrollará el protocolo del objetivo 1, la revisión sistemática (RS) para identificar el conocimiento científico actual sobre la TDC de las mujeres en la DPCM. En los meses 3 y 4, se elaborará una base de datos, se realizará la búsqueda de literatura, se obtendrán los estudios relevantes y se realizará la evaluación de la literatura y de la información obtenida. Asimismo, en los meses 3 y 4 se creará una base de datos y se comenzará el contacto con diferentes ámbitos sanitarios para constituir el comité de expertos. En los meses 5 y 6 se analizarán y sintetizarán los datos, elaborando un informe final durante el siguiente mes (mes 7). Bajo las coordinación del Subproyecto 1, con el apoyo del comité de expertos se desarrollarán los guiones de las entrevistas y grupos focales (mes 7), a la vez que se iniciará el reclutamiento de los participantes para las entrevistas y grupos focales, que se llevarán a cabo en los meses 8, 9 y 10. Paralelamente, durante los meses 7y 8 se obtendrán los datos de costes hospitalarios del tratamiento del cáncer de mama (Hospital del Mar e Institut Català de la Salut). En los meses 9 y 10 se revisarán los costes de detección precoz de los programas de cribado participantes. Los meses 11 y 12 se dedicarán a revisar las bases de datos de empresas consultoras y la bibliografía sobre costes indirectos.

Segundo año: Durante este año se iniciará el ECA para evaluar la efectividad y otras medidas de resultado de la HATD. Durante el 1r trimestre se obtendrá información socio-económica de las áreas básicas de salud (ABS) de los programas de detección precoz participantes. Se seleccionarán las ABS del estudio (primera etapa del muestreo), se obtendrán listados de mujeres que van a ser invitadas a los programas de cribado. Se preparará el algoritmo de selección aleatoria. Se elaborarán los cuestionarios pre-intervención del ECA. Durante el 2º trimestre se colaborará con el Subproyecto 1 en la elaboración de la HATD y se realizará el entrenamiento de los entrevistadores. Paralelamente, durante el 2º trimestre se preparará el estudio de preferencias (objetivo 3).

Se realizará la prueba piloto del ECA (3r trimestre) y se iniciará el ECA (4º trimestre).

Tercer año: El estudio de preferencias se realizará durante el 1r trimestre. El ECA se completará al final del primer semestre. El 3r trimestre se dedicará a analizar los datos del ECA, el estudio de preferencias, y se realizará la evaluación económica y la de daño-beneficio. Finalmente, el último trimestre se dedicará a la elaboración del informe final de este subproyecto.

El personal técnico de apoyo al proyecto tendrá experiencia previa en realización de estudios experimentales poblacionales. Se requerirá formación en metodología de la investigación (salud pública, epidemiología, estadística, informática) y en economía.

El proyecto se coordinará desde el Institut de Recerca Biomédica de Lleida (IRBLLEIDA). Participarán los programas poblacionales de detección precoz del Hospital del Mar, Instituto Catalán de Oncología, Región Sanitaria Lleida, y Canarias.

Expediente Nº
PI14/00113

INVESTIGADOR/A PRINCIPAL: Montserrat Rué Monné

MEMORIA DE SOLICITUD PROYECTO DE INVESTIGACIÓN EN SALUD
SECCIÓN PLAN DE TRABAJO
(Ajústese al espacio disponible. Puede incorporar hasta un máximo de 8 líneas de Actividad/Tarea)

CRONOGRAMA

ACTIVIDAD / TAREA	PERSONA/S INVOLUCRADAS	MESES	E	F	M	A	M	J	J	A	S	O	N	D
Obj.1. Revisión de la literatura. Análisis y síntesis de resultados	MR, MC,LD, MM, MJP, RP, AP, JS, CV	1º Año	✓	✓	✓	✓	✓	✓						
		2º Año												
		3º Año												
Obj. 2 y 3 del Subproyecto 1 en Cataluña<br>Obj. 4. Análisis de costes.	MR, MC,LD, MM, MJP, RP, AP, JS, CV	1º Año							✓	✓	✓	✓	✓	✓
		2º Año												
		3º Año												
Obj. 2 Preparación del ECA.<br>Obj. 3. Preparación del estudio de preferencias.	MR, MC,LD, MM, MJP, RP, AP, JS, CV	1º Año												
		2º Año	✓	✓	✓	✓	✓	✓						
		3º Año												
Obj. 2. Prueba piloto ECA	MR, MC,LD, MM, MJP, AP, CV	1º Año												
		2º Año							✓	✓	✓			
		3º Año												
Obj. 2. ECA-muestra correspondiente a Catalunya. Estudio de preferencias.	MR, MC,LD, MM, MJP, RP, AP, JS, CV	1º Año												
		2º Año										✓	✓	✓
		3º Año	✓	✓	✓	✓	✓	✓						
Obj. 3. Estudio de preferencias. Análisis de datos.<br>Obj. 4. Estudio Coste-efectividad	MR, MC,LD, MM, MJP, RP, AP, JS, CV	1º Año												
		2º Año												
		3º Año							✓	✓	✓			
Elaboración informe final	MR, MC,LD, MM, MJP, RP, AP, JS, CV	1º Año												
		2º Año												
		3º Año										✓	✓	✓

Expediente Nº PI14/00113

INVESTIGADOR/A PRINCIPAL: Montserrat Rué Monné

MEMORIA DE SOLICITUD PROYECTO DE INVESTIGACIÓN EN SALUD
SECCIÓN PLAN DE TRABAJO

Inserte (si lo desea) una imagen con un cronograma.

Expediente Nº PI14/00113

INVESTIGADOR/A PRINCIPAL: Montserrat Rué Monné

MEMORIA DE SOLICITUD PROYECTO DE INVESTIGACIÓN EN SALUD
SECCIÓN EXPERIENCIA DEL EQUIPO

Experiencia del equipo investigador sobre el tema **(Ajústese al espacio disponible)**

El equipo investigador tiene amplia experiencia en investigación en servicios de salud, en las líneas de evaluación de la efectividad y eficiencia de las intervenciones sanitarias, en particular en el área de la detección precoz del cáncer. Es un grupo multidisciplinar con experiencia en investigación clínica y salud pública, evaluación económica, investigación cualitativa y modelización estadística.

La IP es licenciada en Matemáticas y doctora en Economía. Trabaja como investigadora y profesora agregada de Bioestadística. Desde 2005 y en colaboración con R. Pla, M. Carles y MJ Pérez ha trabajado en la modelización de la detección precoz del cáncer de mama (CM). Pertenece al grupo de investigación en Análisis Económico y Salud (GRAES) reconocido por de la Generalitat de Cataluña como grupo consolidado (2014 SGR 978). Forma parte del "Grup de Recerca en Anàlisi Estadística de Supervivència (GRASS)" y de la Red de Investigación en Servicios de Salud y Enfermedades Crónicas (REDISSEC). Ha liderado algunos y participado en múltiples proyectos de investigación clínica y de evaluación de servicios sanitarios.

Misericòrdia Carles es doctora en Economía y profesora de Fundamentos del Análisis Económico en el Dep. de Economía de la Universidad Rovira i Virgili (URV). Actualmente imparte el Curso de Economía de la Salud en el Máster de Ciencias de la Enfermería y en el próximo curso también en el programa de doctorado de Economía y Empresa de la URV. Es responsable del análisis económico de los diferentes proyectos del grupo y coautora de diferentes artículos en economía de la salud. IP del proyecto FIS PI06/90355 y del grupo de investigación GRAES (2014 SGR 978).

Laia Domingo es doctora en Medicina Preventiva y Salud Pública. Está contratada como investigadora post-doctoral por la REDISSEC y forma parte del Servicio de Epidemiología y Evaluación del Hospital del Mar, reconocido por la Generalitat de Cataluña como grupo de investigación consolidado (2014 SGR 427). Ha coordinado y participado en diferentes estudios de investigación centrados en la evaluación de la efectividad del cribado de cáncer de mama, que han derivado en diversas publicaciones y donde ha desarrollado su tesis doctoral.

Montserrat Martínez es licenciada en Estadística y trabaja en la Unidad de Bioestadística y Soporte Metodológico. Es profesora asociada de la Universidad. Ha colaborado, como parte de su doctorado, en la optimización de los programas de cribado de cáncer de mama. En investigación clínica destaca su colaboración como investigadora en marcadores pronóstico del cáncer de tiroides. En investigación básica en cáncer de mama, destaca su colaboración con investigadores de la Washington University.

Mª José Pérez Lacasta es doctora en Economía y profesora titular de la URV en el área de conocimiento de Fundamentos del Análisis Económico del Departamento de Economía. Pertenece al Grupo de Investigación GRAES y es coautora de diversos artículos relacionados con la prestación de servicios sanitarios y la dependencia.

Roger Pla, doctor en Medicina y Cirugía, fue gerente de la Reg. Sanit. Terres de l'Ebre y director del Plan Director de Oncología de Cataluña. Ha dirigido y participado en diversos estudios de evaluación de los resultados de la asistencia oncológica, en particular de la relación entre el volumen y la calidad asistencial. También ha formado parte del equipo evaluador de los Planes de Salud de Cataluña. Fue IP del proyecto FIS PI06/1649 y participa en el grupo de investigación GRAES.

Anna Pons es licenciada en Medicina y actualmente residente de 3º año de Medicina Familiar y Comunitaria en la Región Sanitaria Lleida. Diplomada en enfermería. Ha participado en estudios sobre factores de riesgo cardiovascular, sobre mediación intercultural en centros sanitarios y programa de detección precoz de enfermedades metabólicas.

Jorge Soler es doctor en Medicina y Cirugía, especialista en Medicina Familiar y Comunitaria y profesor de Atención Primaria en la Facultad de Medicina de la Universidad de Lleida. Es coordinador del grupo acreditado de investigación GREDELL destinado al estudio de las desigualdades en salud en Lleida. Tiene amplia experiencia en los estudios sobre desigualdades de salud y en trabajar con sistemas de información sanitarios. Ha participado en el proyecto FIS PI070156 sobre la utilización de los servicios sanitarios en Cataluña en función del origen y del género de los usuarios.

Carmen Vidal es licenciada en Medicina y Cirugía, especialista en Medicina Preventiva y Salud Pública. Es responsable del Programa de detección precoz del cáncer de mama del Instituto Catalán de Oncología. Tiene amplia experiencia en implementación y evaluación de programas de cribado de cáncer. Actualmente forma parte del Comité Asesor del Programa de Cribado de Cataluña y es asesora del Gobierno de Andorra en prevención y detección precoz del cáncer. Ha participado en los proyectos FIS PS09/00790 y PI060386 relacionados con factores de riesgo del cáncer de mama. Es miembro del International Cancer Screening Network.

Expediente Nº PI14/00113

INVESTIGADOR/A PRINCIPAL: Montserrat Rué Monné

MEMORIA DE SOLICITUD PROYECTO DE INVESTIGACIÓN EN SALUD
SECCIÓN MARCO ESTRATÉGICO

(Ajústese al espacio disponible)

1. Capacidad del proyecto de abordar los objetivos, prioridades enmarcadas en el reto Salud, Cambio Demográfico y Bienestar de la Estrategia Española de Ciencia y Tecnología y de Innovación.
2. Capacidad del proyecto de fomentar sinergias e impulsar el talento en el SNS.

1. Los objetivos de este proyecto se enmarcan en las siguientes líneas de investigación prioritarias de la convocatoria AES:

c.2: Calidad, eficiencia, sostenibilidad y solidaridad del SNS
c.10: Participación y empoderamiento de los pacientes ciudadanos
e.4: Innovación en servicios de salud orientada a la mejora de procesos
e.5: Evaluación de servicios sanitarios

El proyecto pretende ampliar el conocimiento sobre los aspectos de participación ciudadana y la viabilidad de nuevos modelos y estrategias en la detección precoz del cáncer de mama. En concreto, pretende indagar y promover la implicación de las mujeres en el proceso de la toma de decisiones, así como la colaboración de ciudadanos y profesionales sanitarios en los modelos y las estrategias de detección precoz del cáncer. Asimismo, se propone explorar la viabilidad de cambiar el paradigma de cribado poblacional actual (bienal entre 50 y 69 años) por un modelo de detección precoz adaptado al riesgo, un cambio ampliamente sustentado por diversos grupos de investigación y programas de cribado internacionales. Los resultados de este estudio aportarán, por tanto, información valiosa y necesaria tanto para el progreso de los actuales programas de cribado, promoviendo una participación ciudadana activa y la corresponsabilidad de la sociedad en la prevención, como para el diseño e implementación eficiente de posibles futuros modelos basados en el riesgo personalizado.

La investigación que se plantea es factible en el marco de colaboración entre el Instituto de Investigación Biomédica de Lleida, los otros centros de investigación participantes y los programas de detección precoz implicados. Se espera publicar estos resultados en revistas internacionales de alto impacto bibliométrico en el campo de la Salud Pública y de la Oncología.

2. Capacidad del proyecto de fomentar sinergias e impulsar el talento en el SNS.

En el proyecto participan 9 investigadores de 8 centros distintos (UdL, IRBLleida, URV, CREIP, ICO-Hospitalet, Parc de Salut Mar, Regió Sanitària Lleida, Institut Català de la Salut) y de perfiles profesionales muy variados (3 economistas, 2 estadísticas, 3 médicos, y 2 matemáticas) y con experiencias en investigación muy diversas. Los objetivos planteados son ambiciosos y los métodos propuestos combinan aproximaciones muy diversas. El proyecto tiene un componente metodológico importante y requiere un trabajo de campo intenso, que requerirá y se beneficiará de la colaboración entre diferentes institutos de investigación y centros sanitarios, localidades y áreas del conocimiento. Esta combinación reforzará la formación de los investigadores participantes y proporcionará resultados relevantes para proponer formas de participación ciudadana en la toma de decisiones.

El proyecto forma parte de un proyecto coordinado bajo la dirección de Montserrat Rué (UdL). Por este motivo, esta propuesta se beneficia de las sinergias que se consiguen con el grupo de investigación liderado por Amado Javier Rivero que es investigador de la Fundación Canaria de Investigación y salud (FUNCIS) que actualmente trabaja para el Servicio Canario de la Salud.

Expediente Nº PI14/00113

INVESTIGADOR/A PRINCIPAL: Montserrat Rué Monné

MEMORIA DE SOLICITUD PROYECTO DE INVESTIGACIÓN EN SALUD
SECCIÓN MEDIOS DISPONIBLES

(Ajústese al espacio disponible)

Medios disponibles para la realización del proyecto

Este proyecto coordinado cuenta con los medios humanos y las instalaciones del SESCS (Canarias), del Instituto de investigación Biomédica de Lleida (IRBLLEIDA), la Facultad de Economía y Empresa de la Universitat Rovira i Virgili y los Programas de detección precoz del cáncer de mama participantes (Canarias, ICO-Hospitalet, Parc de Salut Mar, Regió Sanitària Lleida) donde se dispone de espacios para seminarios, salas de reuniones y salas de actos. Estas instalaciones permiten desarrollar, sin ningún problema, las reuniones de trabajo periódicas, los estudios cualitativos y los talleres o jornadas que se programen en este proyecto. Por lo tanto, se dispone de sus infraestructuras que aseguran un ordenador por técnico superior, impresoras, fotocopiadoras, los programas informáticos necesarios, así como el apoyo técnico que se pueda necesitar procedente de otros departamentos (incluyendo biblioteca de recursos sanitarios y personal documentalista). Además, se dispone de las principales bases de datos bibliográficas (primarias y secundarias) en las que llevar a cabo las búsquedas de los estudios y/o referencias de la evidencia científica: MEDLINE, CINAHL, PsycINFO, etc. Acceso a revistas de interés en el área de epidemiología, salud pública, ETS, oncología, toma de decisiones, etc. Así como el acceso a diversas fuentes de información de interés sanitario. También se cuenta con los software requeridos para el manejo y análisis de los datos R y Stata, así como de los recursos técnicos para la realización de las entrevistas.. Asimismo se cuenta con el apoyo de REDISSEC, del que algunos componentes del equipo son miembros activos.

El grupo investigador cuenta con la asesoría de la Dra. Lilisbeth Perestelo, una de las investigadoras españolas con más experiencia en el diseño y evaluación de las HATD, y mantiene colaboraciones internacionales con reconocidos expertos en el área del cribado poblacional (Dra. Mireille Broeders) y en modelos probabilísticos (profesores Marvin Zelen y Sandra Lee) que apoyarán al equipo de investigación como asesores científicos externos.

Expediente Nº PI14/00113

INVESTIGADOR/A PRINCIPAL: Montserrat Rué Monné

MEMORIA DE SOLICITUD PROYECTO DE INVESTIGACIÓN EN SALUD
SECCIÓN JUSTIFICACIÓN DETALLADA DE LAS PARTIDAS PRESUPUESTARIOS SOLICITADAS
(Ajústese al espacio disponible)

Se trata de un proyecto de investigación que requiere de tres años para su adecuada ejecución. La ayuda solicitada contempla, fundamentalmente, los gastos previsibles en el trabajo de campo (Ensayo Controlado Aleatorizado (ECA) y estudio de preferencias) y la contratación de servicios de personal técnico de apoyo.

La contratación de los servicios de técnicos de apoyo durante los tres años, se justifica por la complejidad del estudio de intervención, así como por la multiplicidad de tareas a realizar. Participación en: búsqueda bibliográfica y RS; contacto, reclutamiento y seguimiento de mujeres y profesionales durante todo el proceso; participación con el subproyecto 1 en el desarrollo de los grupos focales en Cataluña; coordinación del ECA; preparación, validación y coordinación de las entrevistas en los programas de detección precoz de Cataluña; estudio de los costes relacionados con la detección precoz y tratamiento del cáncer según diferentes escenarios de cribado; estudio de preferencias entre cribado uniforme o variable según el riesgo; depuración y análisis de los datos; redacción parcial de los informes; elaboración de publicaciones

El presupuesto necesario para las encuestas telefónicas asistidas por ordenador (sistema CATI) se ha obtenido en base a: Obj. 2: 500 entrevistas pre-intervención (10-15 minutos) y 400 entrevistas post-intervención (20-25 minutos). Obj. 3: 120 entrevistas (20-25 minutos):
- Encuestas por teléfono a mujeres participantes (1020 encuestas): 2100 eur
- Llamadas fallidas: 1400 eur
- Personal entrevistador formado para realizar las encuestas: 11500 eur

En total 15000 eur (6000 2º año y 9000 3r año).

Gastos para el desarrollo de los grupos focales y grupos consultivos: 2100 eur (1r año).

Gastos envío materiales (cartas presentación + envío HATD): total 6000 eur, 2000 el 2º año y 4000 el 3r año.

Acceso a bases de datos privadas de costes sanitarios y no sanitarios: 1.000 eur.

Ordenador portátil para almacenar el trabajo de campo y realizar el análisis estadístico y la evaluación económica: 1850 eur. Asimismo, se necesita una licencia de actualización del programa TreeAge (1r año) para la evaluación económica (500 eur.).

Publicación de tres artículos en revistas libre acceso, uno el 2º año y dos el 3er año (1.300*3): 3.900 eur.

Se solicita presupuesto para los gastos de desplazamientos y dietas de los participantes en el estudio (mujeres invitadas y profesionales, objetivos 2 y 3 del subproyecto 1 en Cataluña), así como del equipo investigador y personal contratado. Se precisa recursos para las reuniones de coordinación a lo largo del proyecto, así como desplazamientos para la presentación y difusión de los resultados.

Difusión y divulgación: Se solicita presupuesto para difundir y presentar los resultados obtenidos en el estudio a través de publicaciones en revistas científicas (traducción de artículos y gastos de publicación), presentaciones en encuentros científicos y congresos a nivel nacional e internacional de Salud Pública, Oncología y Toma de Decisiones Compartidas.

Expediente Nº
PI14/00113

INVESTIGADOR/A PRINCIPAL: Montserrat Rué Monné

MEMORIA DE SOLICITUD PROYECTO DE INVESTIGACIÓN EN SALUD
SECCIÓN PRESUPUESTO

Presupuesto solicitado

**1. Gastos de Personal**	**Euros**
**Subtotal Gastos de Personal :**	

2. Gastos de Ejecución

A) Adquisición de bienes y contratación de servicios
(Bienes inventariable, material fungible y otros gastos)

1er año: Contratación de Servicios (personal técnico de apoyo)	12.000
1er año: Reclutamiento GF y GC, programas de Catalunya (700 euros/programa)	2.100
1er año: Ordenador	1.850
1er año: Información sobre costes (bases de datos consultoría)	1.000
1er año: Actualización programa TreeAge	500
2º año: Contratación de Servicios (personal técnico de apoyo)	15.000
2º año: Encuestas asistidas por ordenador	6.000
2º año: Gastos envío materiales (cartas+HATD)	2.000
2º año: Edición y publicación 1 artículo	1.300
3er año: Contratación de Servicios (personal técnico de apoyo)	15.000
3er año: Encuestas asistidas por ordenador	9.000
3r año: Gastos envío materiales (cartas+HATD)	4.000
3er año: Edición y publicación 2 artículos	2.600
**Subtotal Gastos Bienes y Servicios :**	**72.350**

B) Gastos de Viajes

1er, 2º y 3er año: Desplazamiento y dietas	7.000
2º año: Divulgación de resultados por parte de uno de los miembros del equipo	1.000
3er año: Divulgación de resultados por parte de dos de los miembros del equipo	2.500
**Subtotal Gastos Viajes :**	**10.500**
**Subtotal Gastos Ejecución :**	**82.850**
**Total Presupuesto :**	**82.850**

Expediente Nº PI14/00113

INVESTIGADOR/A PRINCIPAL: Montserrat Rué Monné

MEMORIA DE SOLICITUD PROYECTO DE INVESTIGACIÓN EN SALUD
SECCIÓN ANEXOS

INTRODUZCA TEXTO COMO ANEXO **Máximo 3 páginas.**

Aspectos éticos y limitaciones

Aspectos éticos

En todas las fases del estudio, cuando se seleccione y contacte a los participantes, se les informará por escrito y oralmente de los propósitos, metodología, implicaciones y utilización de las conclusiones del mismo, así como de la posibilidad de abandonar el estudio en cualquier momento. Este estudio no interferirá en el desarrollo de los programas de cribado en lo que se refiere a las futuras invitaciones de las mujeres participantes, sea cual sea su decisión. Se solicitará siempre el consentimiento informado a participar, en el caso de las encuestas telefónicas se enviará por correo pero se solicitará verbalmente en el primer contacto telefónico. Todo el estudio se desarrollará respetando la legislación vigente y convenciones y declaraciones actuales de la Unión Europea sobre reglas éticas de investigación, incluida la Declaración de Helsinki. El proyecto será enviado para su revisión y aprobación a los comités de ética de los programas de detección precoz participantes y del hospital universitario Arnau de Vilanova de Lleida antes de comenzar el trabajo de campo.

Limitaciones

En el estudio intervienen únicamente cuatro programas de DPCM, pudiendo verse comprometida la validez externa de los resultados. Es previsible que el impacto de esta limitación sea reducida dados los objetivos del estudio; aún así se registrarán e intentarán controlar todas aquellas variables que pudieran afectar la validez externa. Con relación a los diseños cualitativos, los grupos focales consumen un tiempo considerable e implican un control menor por parte del investigador, pudiendo limitar la validez y confiabilidad de los resultados. Sin embargo, constituyen la mejor opción cuando se pretende explorar actitudes y evaluar la aceptación de la población de una nueva intervención o un cambio de estrategia, en particular cuando ésta implica su participación expresa.

La asignación del GC y GI se realizará en ABS completas (objetivo 2), lo cual podría sesgar los resultados y limitar asimismo la extrapolación de los datos. Para controlar y evitar al máximo esta limitación, se llevará a cabo una caracterización previa tanto de las ABS como de los individuos, en cuanto a los aspectos socioeconómicos y de resultados de cribado, que se tendrá en cuenta en el muestreo. Las fases del estudio basadas en encuesta pueden generar sesgos de selección (dificultades de contactar a las personas, aceptación a participar asociada a ciertos perfiles, etc) y de información (respuestas socialmente deseables). Todos estos aspectos se tendrán en cuenta tanto en el diseño de la muestra como de los cuestionarios.

Expediente Nº PI14/00113

INVESTIGADOR/A PRINCIPAL: Montserrat Rué Monné

INTRODUZCA IMÁGENES COMO ANEXO Máximo 1 página.

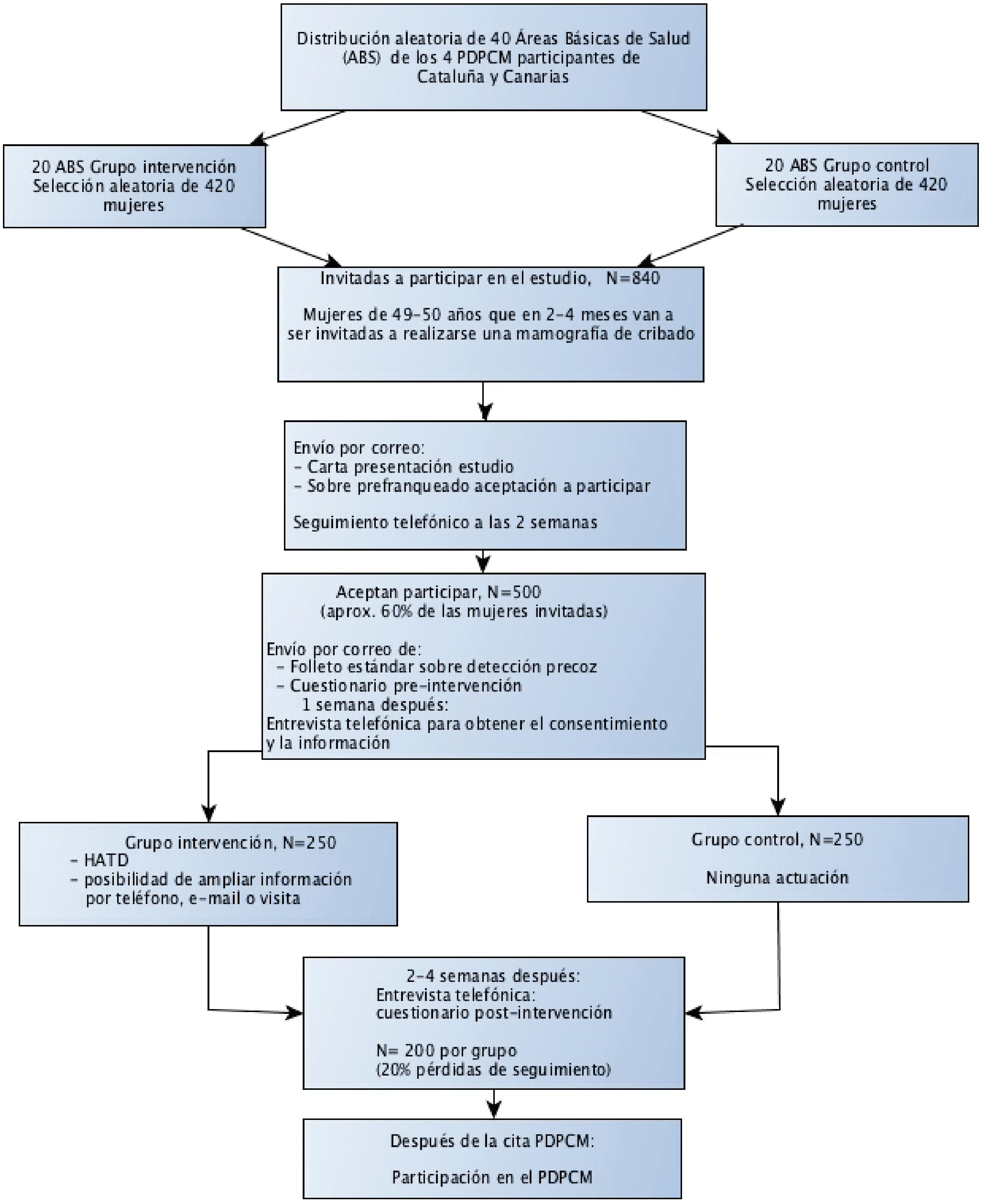

Supplement: S1 File — (PDF) [file pone.0214057.s001.pdf]
