# Supplementary material for: Effect of information about the benefits and harms of mammography on women’s decision making: The InforMa randomised controlled trial
Source: PLoS One. 2019 Mar 26;14(3):e0214057. doi: 10.1371/journal.pone.0214057 (PMC6435150; doi:10.1371/journal.pone.0214057)
Supplement: S2 File — (PDF) [file pone.0214057.s002.pdf]

OBJECTIVES FOR THE WHOLE InforMa PROJECT

The specific objectives of this subproject are:

1) Conduct a synthesis of current scientific knowledge on shared decision making (SDM) in the early detection of breast cancer (EDBC), and more specifically on the development and implementation of decision aids (DA).

2) Evaluate the effectiveness of DA versus usual care, on informed choice, decisional conflict, intention to participate in the EDBC program, and satisfaction.

3) Evaluate the preferences of women between the standard program of EDBC and a program adapted to individual risk.

4)
Conduct cost-effectiveness and harm-benefit analyses of risk-based EDBC compared to uniform EDBC, in a SDM context.

Note: The submitted manuscript refers to Specific Objective 2. We translate the protocol part that corresponds to the submitted work.

PROTOCOL SECTIONS THAT CORRESPOND TO THE RANDOMISED CONTROLLED TRIAL

Design: Experimental study randomized in two stages and controlled. Figure 1 in the annex summarizes the study design.

Study population: Women aged 49-50 years who in 2-4 months will be invited to participate for the first time in the EDBC programs of Parc de Salut Mar (Barcelona), ICO-Hospitalet (Hospitalet de Llobregat), Regió Sanitària Lleida, and Canary Islands. Exclusion criteria: 1) previous history of breast cancer and / or bilateral mastectomy, 2) difficulty of language comprehension, 3) cognitive problems to complete or understand the materials.

Study period: The inclusion period will include the fourth quarter of 2016 and the first semester of 2017. Women will be followed-up until the appointment for the screening exam at the EDBC program.

Pilot test: Before starting the experimental study, a pilot test will be conducted, with 10 women from each participating EDBC program, to verify the suitability of the recruitment and data collection processes. It will take place during the third quarter of 2016.

Selection of women and assignment to the two study groups: In order to reduce intragroup contamination, the first stage of sampling will randomly assign 10 Basic Health Areas (BHAs) or clusters of the 4 EDBC programs participants to the control (CG) and intervention (IG) groups. To maximize the comparability of the CG and IG groups, the BHAs of each EDBC program will be classified according to their socioeconomic characteristics and will be assigned randomly by blocks of size 2 to the CG and IG groups. The information files will be those used by the EDBC program to invite the target population: Central Registry of Insured (RCA) in Catalonia and Registry of the Health Card in the Canary Islands. In the second stage of sampling, 210 women will be randomly selected in each screening program (840 women in total (see section "Sample size")) among those that meet the inclusion criteria, distributed evenly among BHAs of the IG and CG groups. All selected women will be mailed a letter of introduction to the study and a pre-paid envelope with acceptance to participate in the study. A telephone follow-up will be carried out in the interval of two weeks after the shipment to verify that they have received the invitation and evaluate the inclusion/exclusion criteria.

Data collection before the intervention: Women who agree to participate will be mailed the standard information leaflet on early detection of each EDBC program, the pre-intervention questionnaire and a informed consent document. A week later, they will be contacted by telephone to obtain verbal consent and perform the pre-intervention survey. The questionnaire will include sociodemographic variables, previous screening experience, BC risk factors, level

of literacy of medical terms, and use of information and communication technologies.

Intervention: Women of IG will receive by mail the DA in paper format and the web link that contains the DA in web format. They will also be provided with a contact phone and an e-mail to expand the information and solve their doubts. If they want it, they may arrange a face-to-face appointment with a health professional from the research team to reinforce the shared decision making process. Individual interviews with a health professional are not considered because the resources of the EDBC programs are limited and the project aims to assess a realistic scenario. There will be no intervention on women of the CG.

Data collection after the intervention:
The two study groups will be surveyed by telephone again at the end of the intervention period. Women of the IG will be interviewed in the range of 2-4 weeks after having sent the DA and those of the CG in the interval of 2-4 weeks after the pre-intervention interview. A questionnaire will be used that will include knowledge of aspects related to breast cancer and early detection; attitude towards screening; opinion and preferences regarding participation in decision making; intention to participate in the screening program; decision conflict; satisfaction with the decision; and degree of acceptance/utility of the DA (in the GI). Validated scales whose suitability has been demonstrated in previous studies will be used (Hersch 2014, Mathieu 2010). Applications for additional information will be monitored, either by telephone and/or in person, and also the accesses and time of connection to the DA on the web. After the visit corresponding to the first mammography, we will obtain participation in the EDBC program and the reasons for not having participated if applicable.

Main outcome: Informed choice (Hersch 2014), as a dichotomous variable, will be considered the main outcome variable. Following the work of Hersch et al., this variable will be obtained from three components: knowledge, attitudes and intentions. Informed choice is characterized by the intersection of adequate knowledge and consistent attitudes and intentions. Each of the three

components will be measured by a scale that will become dichotomous by means of a “a priori” set cutting point. Adequate knowledge should indicate basic understanding of concepts such as false positives, mortality reduction and overdiagnosis. Attitudes will be measured using a six items scale (Dormandy 2006). Intention to participate or not in the screening will be measured with a 5 categories Likert scale (dichotomized 1-3: no, 4,5: yes). The three components of the main variable will also be analyzed separately.

Secondary outcomes: Participation in screening, decisional conflict (measured with the Decisional Conflict Scale (version of 10 items adapted for the study, O'Connor 1995), satisfaction with the decision, and degree of acceptance/utility of the DA in the IG.

Sample size: The main analysis will compare the proportion of women who make an informed decision in the two study groups using the chi-square test and the confidence interval (95%) of the difference in proportions. We consider an absolute difference of 20% to be relevant. Assuming that the proportion in one of the groups is 50% (most conservative scenario) and an intraclass correlation coefficient equal to 0.1 (clusters sampling), in order to achieve a power of 80% or more to detect a difference between the groups of 20% with a two sided significance level of 5%, 200 women are required per group, 400 in total, 100 per participant program. This sample size is sufficient to detect a difference of 20% in the intentions and a mean difference lower than 0.35 standard deviations in the knowledge and attitudes scales (based on the literature results). Assuming that 60% of the invited women will agree to participate and that there will be 20% lost to follow-up, 210 women will be invited to participate in the study in each EDBC program, 840 women in total.

Statistical analysis: Comparative analysis of the main and secondary outcomes by study groups using confidence intervals and chi-square tests (qualitative variables), Student's t test and Mann-Whitney U test (quantitative variables). Linear and logistic multiple regression models will be used to assess the association between women characteristics and the outcome variables. If the

presence of missing values reduces by more than 10% the number of records of the multivariable models, a multiple imputation method and sensitivity analysis would be used.
