# Supplementary material for: Effect of information about the benefits and harms of mammography on women’s decision making: The InforMa randomised controlled trial
Source: PLoS One. 2019 Mar 26;14(3):e0214057. doi: 10.1371/journal.pone.0214057 (PMC6435150; doi:10.1371/journal.pone.0214057)
Supplement: S4 File — (PDF) [file pone.0214057.s004.pdf]

Estudio InforMa. Cuestionario 1

El estudio **InforMa** es un trabajo de investigación en el que colaboran las Universidades de Lleida, Rovira i Virgili, el Instituto Hospital del Mar de Investigaciones Medicas (IMIM), el Instituto Catalán de Oncología y el Servicio Canario de Salud y que tiene por finalidad:

(A) Evaluar el impacto de una herramienta que ayude a la toma de decisiones compartidas en el proceso de participación en un programa de detección precoz de cáncer de mama.

(B) Evaluar las preferencias de las mujeres en relación a la adaptación del programa de detección precoz al riesgo individual de tener cáncer.

La participación al estudio comporta responder dos cuestionarios. Si acepta participar, le pedimos que conteste este primer cuestionario. Más adelante, nos pondremos en contacto con usted para que conteste al segundo cuestionario. La información recogida en el estudio será totalmente confidencial. Sus respuestas se introducirán en una base de datos protegida que imposibilitará la identificación mediante un sistema de disociación, de acuerdo con la Ley Orgánica 15/1999 de Protección de Datos de Carácter Personal.

Cuando responda las preguntas de los cuestionarios no se preocupe por si las respuestas son correctas o incorrectas. ąąPiense que no está haciendo un examen!!

Si marca una **X** en el recuadro entendemos que da su consentimiento para participar en el estudio: ☐

1. Datos socio-demográficos

1. ¿Cuál es su fecha de nacimiento? (día/mes/año) ___/___/____

2. Lugar de nacimiento

Por favor seleccione **solo una** de las siguientes opciones:

○ En un municipio de Cataluña (especifique el municipio) ____________

○ En un municipio de España, fuera de Cataluña (especifique la provincia) ____________

○ En el extranjero (especifique el país) ____________

3. Lugar de residencia

Por favor seleccione **solo una** de las siguientes opciones:

○ En el mismo municipio de nacimiento

○ En otro municipio de Cataluña (especifíquelo) ____________

4. Nacionalidad

Por favor seleccione **solo una** de las siguientes opciones:

○ Española

○ Española y otra extranjera (especifique el país) ____________

○ Extranjera

5. Año de llegada a España

Contestar esta pregunta solo si se cumplen las siguientes condiciones: Si la respuesta fue 'Española y otra extranjera' o 'Extranjera' en la pregunta de Nacionalidad.

Escriba la respuesta aquí:

____

6. Nivel máximo de estudios finalizados

Por favor seleccione **solo una** de las siguientes opciones:

○ Primarios

○ Educación secundaria obligatoria o equivalente

○ Enseñanzas de bachillerato o FP de Grado Medio

○ Educación Superior (Estudios Universitarios o FP de Grado Superior)

7. Actualmente, ¿cuál es su situación laboral?

Por favor seleccione **solo una** de las siguientes opciones:

○ Trabaja

○ En paro

○ Trabajo en el hogar (ama de casa)

○ Incapacitada

○ Jubilada

○ Otra: ____________

2. Experiencia previa del cribado

8. ¿Realiza alguna de las siguientes actividades de vigilancia de la mama? Responda Sí o No en cada caso

Por favor seleccione **solo una** de las siguientes opciones:

▪ Mamografía	○ Sí	○ No
▪ Exploración física por un profesional sanitario	○ Sí	○ No
▪ Autoexploración	○ Sí	○ No

9. ¿Cuándo se realizó la última mamografía? Conteste esta pregunta **solo** si la respuesta fue 'Sí' en la pregunta '8'. Por favor seleccione **solo una** de las siguientes opciones:

○ Hace menos de 1 año

○ Hace entre 1 año y (menos de) 2 años

○ Hace entre 2 años y (menos de) 3 años

○ Hace 3 años o más

○ No lo recuerdo

10. ¿La última mamografía se la realizó a través....? Conteste esta pregunta **solo** si la respuesta fue 'Sí' en la pregunta '8'.

Por favor, seleccione **solo una** de las siguientes opciones:

○ Del Programa de detección precoz del cáncer de mama (recibió una carta)

○ Del ginecólogo/a del servicio público de salud (CatSalut)

○ De una mutua o médico privado/a

○ Otro: ______________________________

11. ¿Se le ha diagnosticado alguna/as de las siguientes patologías en las mamas?

Por favor, marque las opciones que correspondan:

○ Lesión maligna

○ Lesión benigna

○ Infección

○ No, ninguna

○ Otra: ______________________________

12. ¿Se le ha realizado alguna/as de las siguientes pruebas diagnósticas o tratamientos?

Por favor, marque las opciones que correspondan:

○ Punción quística o PAAF

○ Biopsia

○ Tumorectomía (extirpación de un tumor)

○ Radioterapia

○ Prótesis / Cirurgía estética

○ No, ninguna

○ Otra: ______________________________

3. Factores de riesgo

13. Edad en la primera menstruación

Escriba la respuesta aquí: ________________

14. ¿Cuántos hijos ha tenido?

Escriba la respuesta aquí: ________________

15. ¿A qué edad tuvo el primer hijo?

Conteste esta pregunta **solo** si la respuesta fue mayor que '0' en la pregunta '14'. Escriba la respuesta aquí: ________________

16. ¿Dio de mamar a sus hijos? Conteste esta pregunta **solo** si la respuesta fue mayor que '0' en la pregunta '14'.

Por favor, seleccione **solo una** de las siguientes opciones:

○ Sí

○ No

17. Número de abortos de más de 3 meses Escriba la respuesta aquí: ________________

18. ¿Tiene la menopausia?

Por favor, seleccione **solo una** de les siguientes opciones:

○ Sí

○ No

○ Empiezo a tener síntomas

19. ¿A qué edad tuvo la última regla?

Conteste esta pregunta **solo** si la respuesta fue 'Sí' en la pregunta '18'

Escriba la respuesta aquí: ________________

20. ¿Ha utilizado terapia hormonal para la menopausia (pastillas, parches,...)?

Por favor, seleccione **solo una** de las siguientes opciones:

○ Nunca

○ Sí, menos de 1 año

○ Sí, entre 1 año y (menos de) 3 años

○ Sí, 3 años o más

21. Ha utilizado anticonceptivos orales?

Por favor, seleccione **solo una** de les siguientes opciones:

○ Nunca

○ Sí, menos de 1 año

○ Sí, entre 1 año y (menos de) 3 años

○ Sí, 3 años o más

22. ¿Tiene familiares de primer grado (madre, hermanas, hijas) con cáncer de mama?

Por favor, seleccione **solo una** de les siguientes opciones:

○ Sí

○ No

○ No lo sé

23. ¿Cuántos familiares afectados tiene?

Conteste esta pregunta **solo** si la respuesta fue 'Sí' en la pregunta '22'.

Seleccione **solo una** de las siguientes:

○ Una

○ Dos o más

○ No lo sé

24. ¿Cuál es su peso habitual? (Kg.)

Escriba la respuesta aquí: ________________

25. ¿Y su altura? (cm.)

Escriba la respuesta aquí: ________________

4. Utilización de las TICs

26. Valore de muy fácil (1) a muy difícil (5) las siguientes proposiciones:

Por favor, seleccione la respuesta apropiada para cada campo.

**Me sé mover por el sistema de archivos y programas de mi ordenador y sé los pasos para imprimir documentos**	Muy fácil ○—○—○—○—○ Muy difícil
**Utilizo las herramientas de navegación por Internet, el correo electrónico y soy capaz de descargar recursos**	Muy fácil ○—○—○—○—○ Muy difícil
**Utilizo herramientas de comunicación social: chats, fórums, redes sociales o mensajería instantánea**	Muy fácil ○—○—○—○—○ Muy difícil

5. Conocimiento general

Valore las siguientes frases en una escala del 1 (muy mala) al 5 (muy buena)

**27.**	**Mi comprensión sobre las ventajas de la mamografía de cribado es...**	Muy mala ○—○—○—○—○ Muy buena
**28.**	**Mi comprensión sobre los riesgos y los posibles efectos adversos de la mamografía del cribado es...**	Muy mala ○—○—○—○—○ Muy buena

6. Actitudes generales

¿Cómo son de relevantes los riesgos y los beneficios potenciales de la mamografía de cribado para usted? Valore las siguientes frases en una escala del 1 (nada importante) al 5 (muy importante)

**29.**	**Para mí, conocer los beneficios de la mamografía de cribado es....**	Nada importante ○—○—○—○—○ Muy importante
**30.**	**Para mí, conocer los riegos de la mamografía de cribado es....**	Nada importante ○—○—○—○—○ Muy importante

7. Intenciones generales

¿Cuál es su opinión? Valore las siguientes frases en una escala del 1 al 5.

**31.**	**Para mí, personalmente, mi participación en el cribado mamográfico es...**	Nada acertada ○—○—○—○—○ Muy acertada
**32.**	**Para mí, personalmente, mi participación en el cribrado mamográfico sería...**	Nada importante ○—○—○—○—○ Muy importante
**33.**	**Para mí, personalmente, mi participación en el cribrado mamográfico sería...**	Nada desagradable ○—○—○—○—○ Muy desagradable

8. Alfabetización sanitaria

34. En una escala de "muy fácil" a "muy difícil", indique cuál es el grado de dificultad que tendría para realizar las siguientes actividades:

	1. Muy fácil	2. Fácil	3. Difícil	4. Muy difícil	5. No sé
**1. Encontrar información sobre los tratamientos asociados a las enfermedades que son de su interés.**	○	○	○	○	○
**2. Averiguar dónde conseguir ayuda profesional cuando se encuentra enfermo (por ejemplo: un doctor/a, farmacéutico/a o psicólogo/a).**	○	○	○	○	○
**3. Entender lo que me dice el doctor/a.**	○	○	○	○	○
**4. Entender las instrucciones del personal médico o de farmacia sobre cómo tomar las medicinas recetadas.**	○	○	○	○	○
**5. Valorar cuándo puede necesitar una segunda opinión de otro doctor/a.**	○	○	○	○	○
**6. Utilizar la información proporcionada por el personal médico para tomar decisiones sobre su enfermedad.**	○	○	○	○	○
**7. Seguir las instrucciones de su doctor/a o farmacéutico/a.**	○	○	○	○	○
**8. Encontrar información sobre la manera de abordar problemas de salud mental como el estrés o la depresión.**	○	○	○	○	○
**9. Comprender las recomendaciones sanitarias relacionadas con hábitos como fumar, hacer poco ejercicio físico o beber alcohol en exceso.**	○	○	○	○	○
**10. Comprender por qué necesita hacerse pruebas de detección precoz de enfermedades o chequeos médicos (por ejemplo: mamografía, prueba de nivel de azúcar en sangre y tensión arterial).**	○	○	○	○	○
**11. Valorar la fiabilidad de la información sobre riesgos para la salud que aparece en los medios de comunicación (por ejemplo: TV, Internet u otros medios de información).**	○	○	○	○	○
**12. Decidir cómo protegerse de las enfermedades gracias a la información que proporcionan los medios de comunicación (por ejemplo: periódicos, folletos, Internet u otros medios de información).**	○	○	○	○	○
**13. Encontrar actividades que sean buenas para su bienestar mental (por ejemplo: meditación, ejercicio físico, paseos, etc.).**	○	○	○	○	○
**14. Comprender los consejos sobre salud que dan la familia y los amigos.**	○	○	○	○	○
**15. Comprender la información proporcionada por los medios de comunicación sobre cómo mejorar su salud (por ejemplo: Internet, periódicos, revistas, etc.)**	○	○	○	○	○
**16. Valorar cuáles de sus hábitos diarios afectan a su salud (por ejemplo: costumbres relacionadas con el consumo de alcohol, hábitos alimenticios, ejercicio físico, etc.).**	○	○	○	○	○

35. En el recuadro siguiente puede escribir los comentarios o dudas que tenga sobre el estudio o las preguntas del cuestionario.

¡Muchas gracias por su colaboración!

GOBIERNO DE ESPAÑA
MINISTERIO DE ECONOMÍA Y COMPETITIVIDAD
Instituto de Salud Carlos III
Unión Europea
Fondo Europeo de Desarrollo Regional
"Una manera de hacer Europa"
