# Supplementary material for: Effect of information about the benefits and harms of mammography on women’s decision making: The InforMa randomised controlled trial
Source: PLoS One. 2019 Mar 26;14(3):e0214057. doi: 10.1371/journal.pone.0214057 (PMC6435150; doi:10.1371/journal.pone.0214057)
Supplement: S5 File — (PDF) [file pone.0214057.s005.pdf]

DO NOT FORGET THAT ...

Mammography does not prevent you from having breast cancer. In addition, it is not a perfect method; some tumors are very difficult to see in a mammogram. You may not have cancer. But if you had it, the diagnosis and treatment at an early stage of the tumor increases the likelihood of survival.

Although you have had a mammogram recently, it is important that if you notice any change in your breasts, go to your doctor.

The information presented in this brochure has been based on scientific articles and materials developed by the National Health Service in England, the Cochrane Collaboration and screening programs in Catalonia.

GOBIERNO DE ESPAÑA
MINISTERIO DE ECONOMÍA Y COMPETITIVIDAD
Instituto de Salud Carlos III
Unión Europea
Fondo Europeo de Desarrollo Regional
"Una manera de hacer Europa"

Study PI14/00113: Women's participation in decisions and strategies for early detection of breast cancer.

Cofunded by Fondo Europeo de Desarrollo Regional (FEDER) of the EU. Participants: Lleida Biomedical Research Institute-University of Lleida, University Rovira i Virgili, Catalan Institute of Oncology, Hospital del Mar and Canary Islands Health Service.

LONG-TERM BENEFITS AND HARMS OF EARLY DETECTION OF BREAST CANCER

If a group of 200 women between 50 and 69 years of age are screened with mammography every 2 years, when they are 80 years old ...

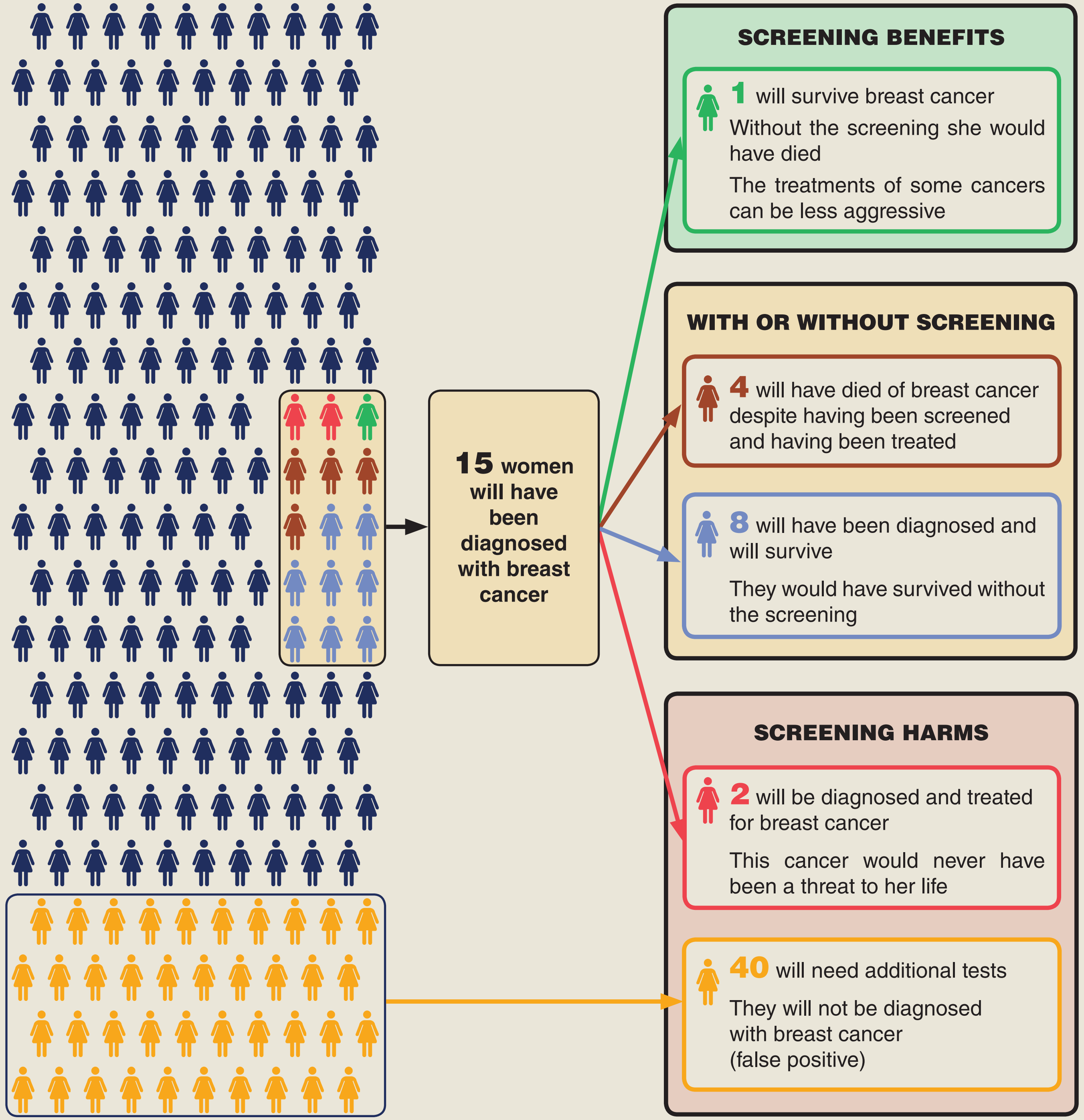

For each death avoided by the screening program, two women can be diagnosed and treated for a cancer that would never have put their life at risk

EARLY DETECTION OF BREAST CANCER

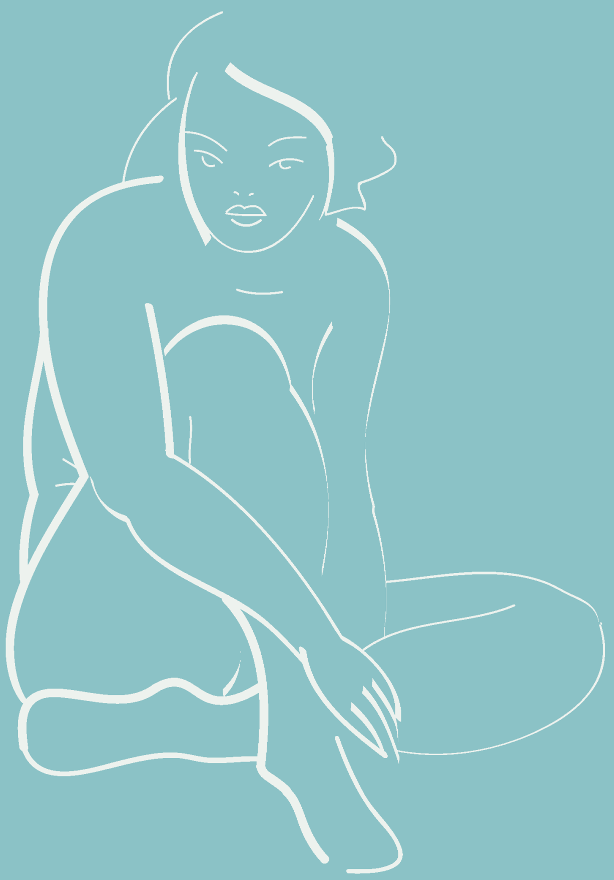

HELPING YOU DECIDE

PARTICIPATE OR NOT PARTICIPATE IN THE SCREENING FOR BREAST CANCER: THIS IS THE QUESTION

Recent scientific studies have identified harms, previously unknown, associated with early detection of breast cancer by mammography. The purpose of this brochure is to inform you about the potential benefits and harms of participating in a breast cancer early detection program.

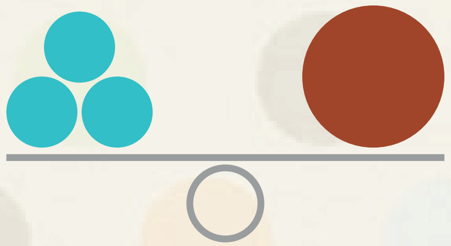

This informative material aims to help you weigh the pros and cons in your personal decision about whether or not you want to participate in the breast cancer early detection program, based on your own values and preferences.

WHAT IS BREAST CANCER?

Breast cancer develops when some cells begin to grow uncontrollably, forming a tumor. As the tumor grows the malignant cells can move to other parts of the body and endanger the life of the affected person.

In Catalonia, about 4,000 new cases of breast cancer are diagnosed every year. According to the statistics, 1 out of every 9 women will suffer breast cancer during their lives and 83% of the women affected will survive this illness.

WHAT IS EARLY DETECTION OF BREAST CANCER?

Early detection of breast cancer, also called screening, is aimed at detecting breast cancer at a very early stage, before it causes symptoms. In this initial stage, cancer is easier to treat and the chances of survival are higher.

The public health system offers the possibility of participating in the breast cancer early detection program in order to reduce the mortality caused by this tumor. The screening program is aimed at women between 50 and 69 years of age and involves mammography every two years.

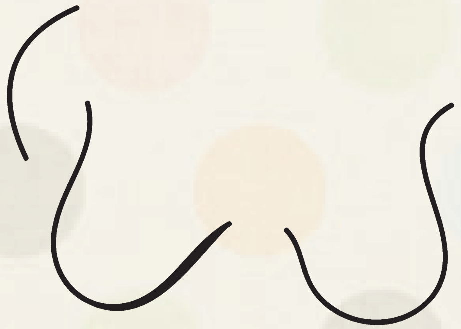

Mammography is an X-ray of the breast. It is the most effective test to detect breast cancer in women who have no symptoms. The risk of being damaged by exposure to this radiation is very small. Some women may need other tests if the mammogram shows an abnormal image.

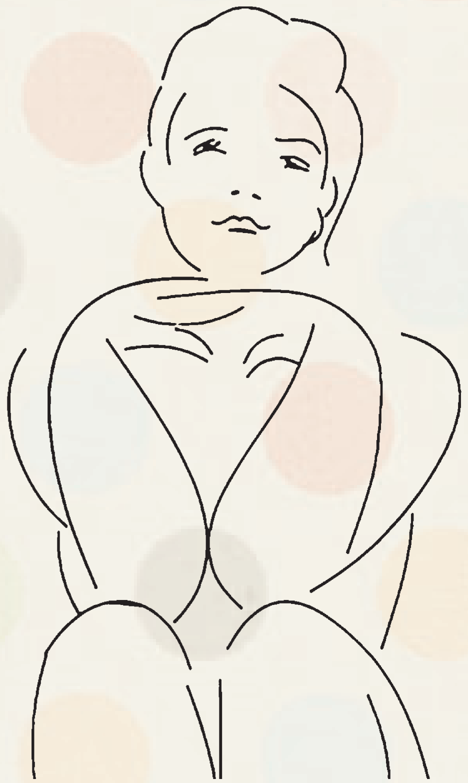

BENEFITS OF SCREENING

Screening reduces the risk of dying of breast cancer

Early detection can save some women's lives because they are diagnosed and treated before they would have been without screening.

Of every 200 women who are screened every two years between 50 and 69 years of age, 1 woman is saved thanks to the early detection of the tumor.

Screening detects cancer in more initial stages

Cancer detected in early stages does not require treatments as aggressive as when it is more advanced; these treatments have fewer side effects and the likelihood of recovery is higher.

ADVERSE EFFECTS OF SCREENING

Errors in diagnosis: false positives and false negatives

False positives occur when mammography results suggest a possible breast cancer that does not really exist. This entails additional explorations that would not be necessary.

Of every 200 women who are screened every two years between 50 and 69 years of age, 40 will have a false positive result.

The opposite situation, the false negative, is much less frequent and occurs when the mammogram does not show any signs of the disease, even if the woman suffers it.

Screening can detect harmless tumors

Some types of cancer that are detected by screening mammography grow so slowly that they would never become a health problem.

Some, even, would disappear spontaneously, without treatment.

Currently, one can not know which tumors would progress and which ones would not, and therefore, treatment is offered to all women diagnosed. Some women may receive treatments that have important side effects, without needing them. This is known as **overdiagnosis** and **over treatment**.

Of every 200 women who are screened every two years between 50 and 69 years of age, 2 will be treated of cancer without need.
