# Supplementary material for: Effect of information about the benefits and harms of mammography on women’s decision making: The InforMa randomised controlled trial
Source: PLoS One. 2019 Mar 26;14(3):e0214057. doi: 10.1371/journal.pone.0214057 (PMC6435150; doi:10.1371/journal.pone.0214057)
Supplement: S6 File — (PDF) [file pone.0214057.s006.pdf]

Un Programa per a detectar el càncer de mama, molt abans que es manifesti.

El càncer de mama és el tumor més freqüent en les dones i **el risc de patir-lo augmenta amb l'edat**. Si es detecta a temps, té moltes probabilitats de curació.

Avui en dia, es curen el 85% dels càncers de mama que es detecten quan el tumor encara està poc desenvolupat.

El Departament de Salut, a través del Programa de detecció precoç del càncer de mama, et convida –si tens entre 50 i 69 anys- a fer-te una mamografia gratuïta cada dos anys.

A partir dels 70 anys les dones han de seguir amb les revisions que els indiqui el seu metge.

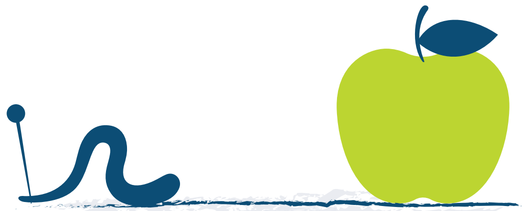

Un Programa para detectar el cáncer de mama, antes de que se manifieste.

El cáncer de mama es el tumor más frecuente en las mujeres , el riesgo de padecerlo aumenta con la edad. Si se detecta a tiempo, tiene muchas probabilidades de curación.

Actualmente, se curan el 85% de los cánceres de mama que se detectan cuando el tumor todavía está poco desarrollado.

El Departament de Salut a través del Programa de Detección Precoz del Cáncer de Mama, te invita -si estás entre los 50 y 69 años- a hacerte una mamografía gratuita cada dos años.

A partir de los 70 años todas las mujeres deben continuar con las revisiones indicadas por su médico.

En cas de descobrir alguna anomalia als teus pits, no dubtis en consultar el teu metge.

Tot i que t'hagis fet una mamografia recentment, **cal consultar el teu metge sempre que detectis alguna anomalia als pits: alteracions de qualsevol tipus, canvi de color o de textura de la pell, presència de bonys, dolor, secreció de líquids, canvi de mida o de forma...**

Per reduir el risc de patir un càncer, a més a més de fer-te mamografies, és important que segueixis alguns consells:

- Practica exercici físic amb regularitat
- Controla el teu pes
- Limita el consum d'alcohol
- Menja 5 racions de fruita i verdura al dia com a mínim.

En caso de descubrir alguna anomalía en tus pechos, no dudes en consultar a tu médico.

Aunque te hayas hecho una mamografía recientemente, debes consultar a tu médico siempre que detectes alguna anomalía en los pechos: alteraciones de cualquier tipo, cambio de color o de textura de la piel, presencia de bultos, dolor, secreción de líquidos, cambio de volumen o de forma ...

Para reducir el riesgo de padecer un cáncer, además de hacerte mamografías, es importante que sigas algunos consejos:

- Practica ejercicio físico con regularidad
- Controla tu peso
- Limita el consumo de alcohol
- Toma al menos 5 raciones de fruta y verdura fresca al día

Per quedar-te tranquil·la, participa-hi; tu decideixes.

Properament rebràs una carta del Programa de Detecció Precoç del Càncer de Mama amb la data, l'hora i el lloc per fer-te una mamografia.

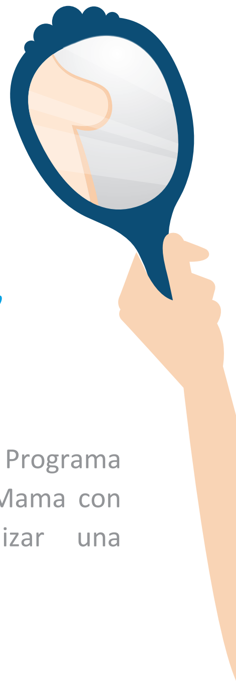

Para quedarte tranquila, participa; tu decides.

Próximamente recibirás una carta del Programa de Detección Precoz del Cancer de Mama con fecha, hora y lugar para realizar una mamografía.

La teva tranquil·litat, bé val una petita molèstia

Per aconseguir mamografies de bona qualitat és necessari comprimir el pit uns segons, la qual cosa pot causar una mica de dolor.
Per evitar radiacions innecessàries, és convenient mantenir un interval de dos anys entre mamografies.

Tu tranquilidad, bien vale una pequeña molestia.

Para conseguir mamografías de buena calidad es necesario comprimir unos segundos el pecho, lo que puede causar algo de dolor.
Para evitar radiaciones innecesarias, es conveniente mantener un intervalo de dos años entre las mamografías.

Amb una mamografia cada dos anys,

el càncer de mama es pot agafar a temps.

La mamografia és una radiografia del pit que permet diagnosticar el càncer de mama quan encara és tan petit que no es nota al palpar-lo. Així, es pot interrom-pre l'evolució de la malaltia i ajuda que el tractament sigui menys agressiu.

A vegades -a més a més de la mamografia- cal fer proves addicionals per descartar o confirmar possibles anomalies. El Programa vetlla per tal que les proves es realitzin en el terminis i amb la qualitat adequada.

Con una mamografía cada dos años,

el cáncer de mama se puede coger a tiempo.

La mamografía es una radiografía del pecho que permite diagnosticar el cáncer de mama cuando todavía es tan pequeño que no se aprecia al tacto. De esta manera se puede interrumpir la evolución de la enfermedad y ayuda a que el tratamiento sea menos agresivo.

En algunas ocasiones –junto con la mamografía- es necesario realizar otras pruebas para descartar o confirmar posibles anomalías. El Programa controla que tales pruebas se efectuen en los plazos correctos y con la calidad adecuada.

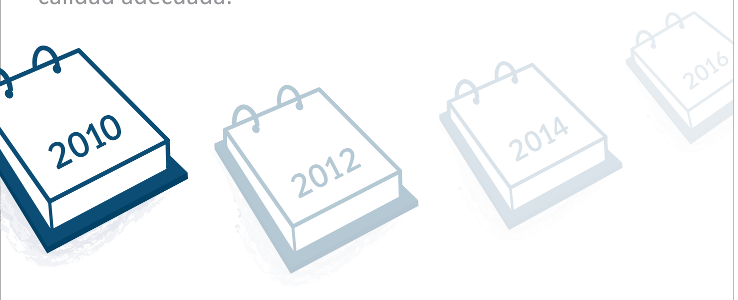

Estudi PI14/00113 Participación de las mujeres en las decisiones y estrategias de detección precoz del cáncer de mama. Cofinançat pel Fondo Europeo de Desarrollo Regional (FEDER) de la UE. Participen: Institut de Recerca Biomèdica de Lleida-Universitat de Lleida, Universitat Rovira i Virgili, Institut Català d'Oncologia, Institut Hospital del Mar d'Investigacions Mèdiques (IMIM) i Servicio Canario de Salud.

Unión Europea
Fondo Europeo de Desarrollo Regional
"Una manera de hacer Europa"

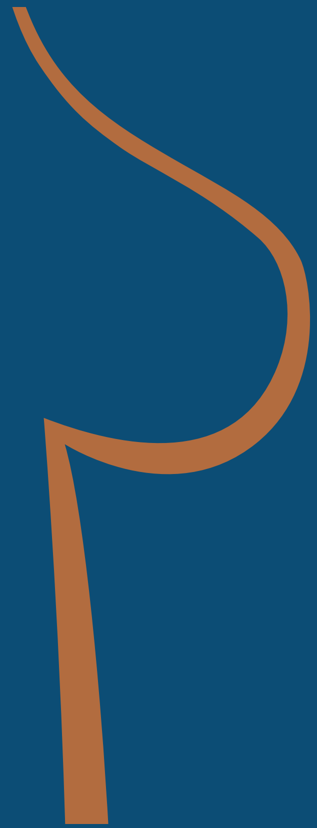

A partir dels 50,

per preveure el càncer de mama

cal que et facis una mamografia cada 2 anys.

Coneix-ne els avantatges
